# Work ability and return-to-work of patients with post-COVID-19: a systematic review and meta-analysis

**DOI:** 10.1186/s12889-024-19328-6

**Published:** 2024-07-07

**Authors:** Marcel Ottiger, Iris Poppele, Naveen Sperling, Torsten Schlesinger, Katrin Müller

**Affiliations:** Institute of Human Movement Science and Health, Faculty of Behavioral and Social Sciences, Chemnitz University of Technology, 09107 Chemnitz, Germany

**Keywords:** Post-COVID-19, Work ability, Return-to-work, Sick leave, Occupational status

## Abstract

**Background:**

In addition to several sequelae of post-COVID-19, individuals also experience significant limitations in work ability, resulting in negative consequences for the return-to-work (RTW) process. This systematic review and meta-analysis were conducted to assess the impact of post-COVID-19 on work ability and RTW of individuals previously infected with SARS-CoV-2.

**Methods:**

Studies on the work ability and RTW of patients with post-COVID-19 (more than 12 weeks after an acute SARS-CoV-2 infection) were regarded eligible for inclusion. Systematic search of literature was performed up to March 2023 using five databases (MEDLINE, EMBASE, CINAHL, CENTRAL and WHO COVID 19). Study selection followed the Preferred Reporting Items for Systematic Review and Meta-analysis (PRISMA) Statement. A meta-analysis estimated the overall success rate of RTW. The risk of bias of the included studies was evaluated with the Newcastle Ottawa Scale (NOS).

**Results:**

19 relevant studies, published between 2021 and 2023, were included in the systematic review, involving 21.155 patients from 14 different countries. The findings indicate that a significant proportion of individuals with post-COVID-19 experience persistent symptoms and functional impairments, with fatigue being the most prominent symptom. These persistent symptoms can have a considerable (negative) impact on individuals’ physical and psychological capacity to participate in work-related activities, leading to lower work ability and increased absenteeism. The RTW for post-COVID-19 patients is complex, with approximately 60.9% of patients successfully returning to work after 12 or more weeks following SARS-CoV-2 infection. Among those who successfully returning to work, a considerable number need modifications in their work duties or hours to cope with residual impairments. Factors such as workplace accommodations, supportive policies, and occupational rehabilitation programs play a crucial role in facilitating successful RTW.

**Conclusions:**

The systematic review underscores the substantial impact of post-COVID-19 on work-related outcomes. The implications of this research highlight the need for healthcare providers, employers, and policymakers to collaborate in creating inclusive work environments and implementing tailored rehabilitation programs to support individuals recovering from post-COVID-19. Further research should focus on long-term follow-up studies with mixed methods to gain a more comprehensive understanding of the long-term consequences of post-COVID-19 on work ability and RTW outcomes.

**PROSPERO registration number:**

CRD42023385436.

**Supplementary Information:**

The online version contains supplementary material available at 10.1186/s12889-024-19328-6.

## Background

Workplaces were generally a high-risk setting for virus transmission of SARS-CoV-2 due to interpersonal contacts with colleagues, clients or patients [1, 2]. Reuter et al. [3] conducted a study involving more than 100.000 workers across diverse occupational segments such as medical healthcare, as well as business management, and observed an incidence rate of 3.7 infections per 1.000 workers. SARS-CoV-2 infections were higher in essential (180 infections among 33.458 workers) workers compared with workers in non-essential (224 infections among 75.502 workers) occupations (incidence rate ratio 1.95) [3]. Particularly, healthcare workers were more likely to be affected by COVID-19 (coronavirus disease 2019), compared with other professions [4–6]. In Germany until October 2023, 350.045 cases of COVID-19 were recognized as occupational diseases (BK) with BK-No. 3101. Furthermore, 26.698 recognized cases of COVID-19 were recorded as work-related accidents (according to the German Social Accident Insurance) [7]. Acute infection with SARS-CoV-2 can lead to several persistent symptoms [8–10]. According to the NICE (National Institute for Health and Care Excellence) guideline, persistent signs and symptoms after an acute SARS-CoV-2 infection, which continue for more than 12 weeks and cannot be explained by an alternative diagnosis, are classified as post-COVID-19 [11].

In population-based cohort studies, the prevalence of ongoing post-COVID-19 symptoms was estimated to be around 6% depending on, for example, virus variants, study design and study population [12–14]. A pooled analysis of data from 22 countries defined three main post-COVID-19 symptom clusters: persistent fatigue, cognitive problems and ongoing respiratory problems [12]. This study showed, that 6.2% of over one million individuals, who had symptomatic SARS-CoV-2 infection, experienced at least 1 of the 3 symptom clusters [12]. In a systematic review, including 70 studies of working age adults, the most frequently reported long-/post-COVID-19 symptoms were fatigue (92%), shortness of breath (82%), muscle pain (44%), and joint pain (35%) [15]. Moreover, findings from systematic reviews and meta-analyses indicated, that the prevalence of post-COVID-19 symptoms in adults, who were hospitalized due to COVID-19, was substantially higher, than in cases with mild or asymptomatic courses of the disease [16, 17]. Studies revealed that both physical as well as neuropsychological limitations can persist for several months (4–24 months) after acute SARS-CoV-2 infection [18–22]. Especially in the case of post-infectious fatigue, a symptom that affected a large number of post-COVID-19 patients, results on long-term courses of other viral and non-viral infectious diseases (e.g., SARS virus, Q-fever, Lyme disease) indicated the risk of chronification [23].

Patients also reported severe limitations in their ability to work with negative consequences on the RTW process. A systematic review concluded that long- and post-COVID-19 symptoms are increasing problems in occupational medicine, because they influence the RTW-process and quality of life of workers previously hospitalized with SARS-CoV-2 [24]. Even a mild SARS-CoV-2 infection can result in a significant reduction in work capacity [25]. Work ability is a multidimensional concept of various factors that enable employees to successfully complete the work tasks [26]. The interaction of both personal resources (health, psychophysical performance, professional competence, values and attitudes) as well as work requirements (e.g., work conditions) for maintaining individual work ability takes place in the concept of the “House of Workability” [27]. Previous research showed that a variety of factors influence the work ability of people with long-term diseases, such as work demands, age, gender, comorbidities and somatic complaints [28]. In addition, poor work ability was also associated with early retirement [29], a factor that had significant consequences for both the labor market (fewer skilled workers) and the economy (low productivity) [30]. As we delved into the concept of RTW, this comprehensive understanding became crucial. Importantly, the RTW process hinged on the restoration of work ability, emphasizing the need for employees to recover their physical and mental capacity to work before entering the occupational reintegration phase. Previous studies showed that improved work ability influenced the RTW positively [31, 32]. This relation emphasized the interdependence of work ability and the subsequent RTW, which refers to the process of reintegration into the workforce after an extended period of absence, whether due to illness, injury, or other reasons. Accordingly, various factors both at individual (e.g., socioeconomic status, expectations, psychological recourses) as well organizational level (e.g., workplace factors, RTW coordination) can determine successful RTW after an injury or illness [33]. In the case of post-COVID-19 patients, the RTW process might be complicated by a range of factors, such as ongoing symptoms, and long-term effects of the disease [34]. Aben et al. [35] observed variations in the RTW duration influenced by different virus variants. The duration was found to be longest when the alpha variant was predominant and became progressively shorter with the emergence of the delta and omicron variants [35]. As highlighted by the meta-analysis of Kamdar et al. [36], conducted before the COVID-19 pandemic, RTW after critical illness is often delayed (36% at 1–3 months, 60% at 12 months) and accompanied by worsening employment status and performance (e.g., fewer work hours).

While the long-term physical and psychological effects of COVID-19 are meanwhile well documented, the potential occupational impact remains to be explored. Understanding the work ability and RTW of patients with post-COVID-19 is crucial for healthcare professionals, occupational health professionals, employers, and policymakers in adapting or developing strategies and interventions to support the recovery and reintegration of these patients into the workforce and to ensure their social participation. It is particularly pivotal for mitigating potential increases in occupational disability and early retirements, thereby playing an essential role in minimizing broader impacts on the labor market and the economy. However, there is currently limited research available on this topic. A systematic review of the available evidence on work ability and RTW of patients with post-COVID-19 is therefore needed to provide a comprehensive analysis of the existing literature and to identify gaps in knowledge and research needs that guide future research. The aim of this systematic review and meta-analysis is to synthesize the evidence on work ability and RTW of patients with post-COVID-19. Specifically, the review will address the following research question:

What is the impact of post-COVID-19 on work ability and the RTW process of patients previously infected with SARS-CoV-2?

By addressing and bridging the gaps in knowledge and synthesizing the available evidence, this systematic review will contribute to systematize the growing body of literature on the post-COVID-19 population and support efforts to mitigate the long-term effects of the pandemic on the global workforce.

## Methods

This systematic review of the literature was conducted using the Preferred Reporting Items for Systematic Reviews and Meta-Analyses (PRISMA) Statement [37]. To ensure transparency and reproducibility, the review adheres to the PRISMA checklist, which can be found in Appendix 1 in the supplementary materials. The protocol was registered in the International Prospective Register for Systematic Reviews (PROSPERO) database (Registration number: CRD42023385436).

### Search strategy

The PRISMA Statement suggest performing the search across multiple databases; therefore, we have chosen five databases due to their relevance in the medical field. A comprehensive search in MEDLINE (via EBSCO), EMBASE (via Ovid), CINAHL (via EBSCO), Cochrane Central Register of Controlled Trials (CENTRAL) and WHO COVID 19 was conducted from January 2020 until December 2022 to encompass the period since the onset of the COVID-19 pandemic. The search in the five databases was repeated in March 2023 to identify further studies with longer analysis periods. The literature search strategy used MeSH terms (Medical Subject Headings) and text words associated with post-COVID-19 and work ability or RTW. MeSH terms used to perform the search in MEDLINE, were the following:

(“coronavirus” OR “covid-19” OR “sars-cov-2” OR “coronavirus infections” OR “betacoronavirus”) AND (“workplace” OR “return to work” OR “absenteeism” OR “occupational health” OR “work performance” OR “work capacity evaluation” OR “sick leave”).

SIGN search filters were used to identify randomized trials and observational studies in the MEDLINE, CINAHL and EMBASE databases [38]. In addition, secondary searches in other sources, such as Google Scholar and medRxiv were also carried out, to retrieve relevant publications that were not found with the database search. To ensure that the literature was comprehensive, the reference lists of the included studies or relevant reviews identified through the search, were scanned. Furthermore, a search was carried out in the German Register for Clinical Studies (DRKS) and the International Clinical Trials Registry Platform (ICTRP) search portal of the WHO to identify ongoing, discontinued and completed studies. The full search strategy is presented in Appendix 2. The literature search was conducted independently by two reviewers.

### Eligibility criteria

Studies on the work ability and RTW of patients with post-COVID-19 were regarded eligible for inclusion if the following criteria were fulfilled: (1) population: patients with persistent signs and symptoms more than 12 weeks after an acute SARS-CoV-2 infection; (2) intervention: SARS-CoV-2 infection (diagnosed by RT-PCR, suspected, self-report). Comparison was not applicable due to the aim of the performed review; (3) outcomes: the primary outcome measures of the systematic review were work ability and RTW of patients with post-COVID-19. Since work ability and RTW can be measured in different ways, several methods for outcome collection were accepted for this review, including for example interviews; and (4) following types of studies: interventional studies (e.g., randomised clinical trials) and observational studies (e.g., prospective cohort studies).

The exclusion criteria were as follows: (1) studies involving subjects without SARS-CoV-2 infection; (2) case series, case reports, pilot studies, unpublished data, editorials, news articles, commentaries, studies not involving humans and systematic reviews; and (3) studies published before 2020. This review was restricted to articles written in English or German language.

### Study selection

**Fig. 1:**
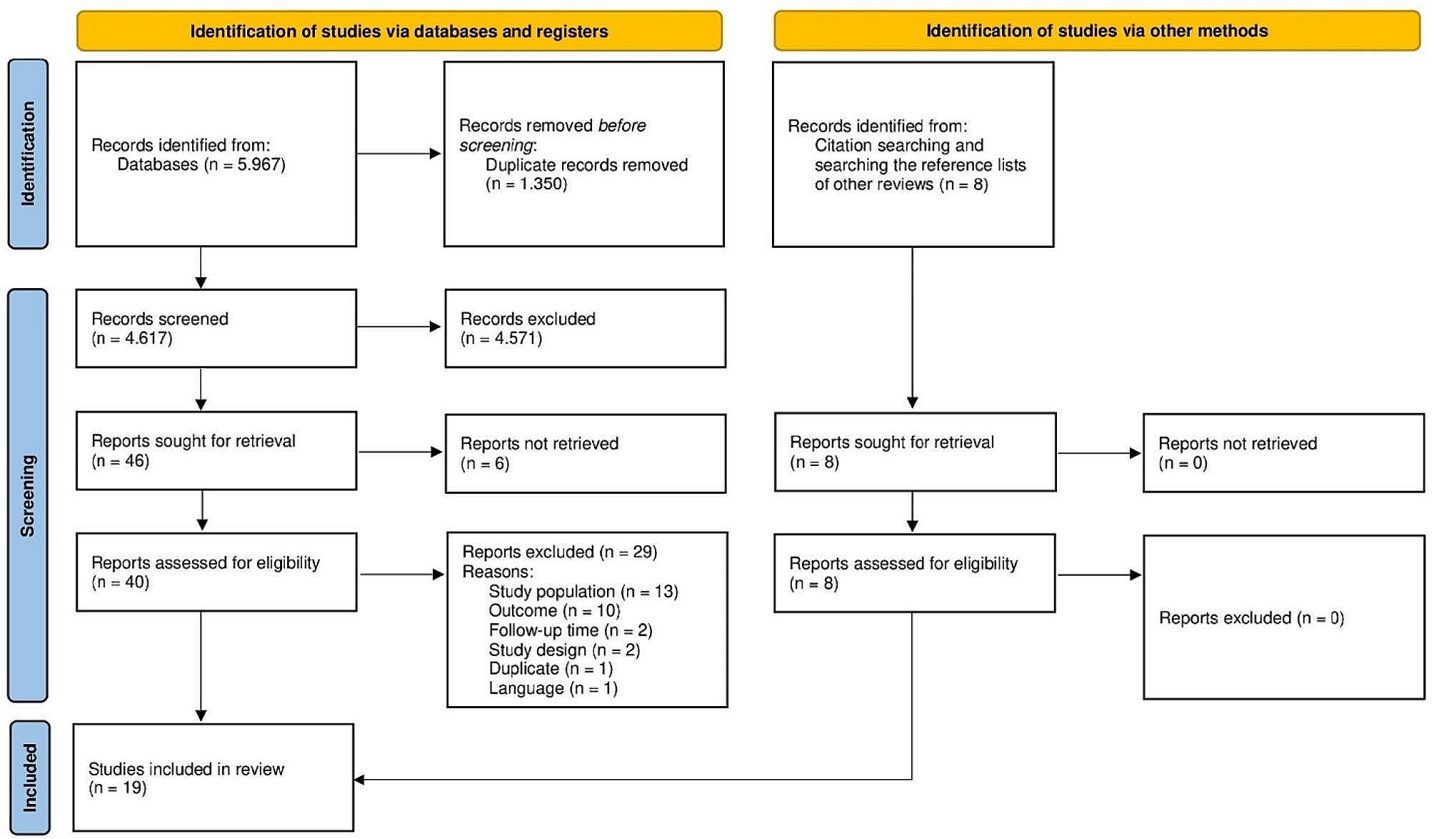
PRISMA flowchart of the article selection process

The screening of articles was carried out in two stages [39]. After duplicates were removed, two persons independently screened the titles and abstracts of references retrieved from the searches and categorized them as relevant, not relevant, or possibly relevant. Studies that clearly did not align with the research objectives, such as those unrelated to COVID-19 work outcomes, were excluded. Studies that were categorized as relevant or potentially relevant by at least one reviewer underwent a full-text-screening. In this second stage the full-text versions of eligible articles were evaluated by both reviewers regarding the inclusion criteria independently. Articles were included if they met the predefined criteria. Any disagreement for inclusion was resolved through discussion between the reviewers and, if necessary, via consultation of the supervising researcher. The screening process was facilitated using Rayyan [40], which allowed blinding in each step of the process. The selection process is documented in the PRISMA flowchart (Fig. 1).

### Data extraction

At the end of the filtering, the extracted features were recorded using a pre-designed data table in Excel. The review authors extracted data from each eligible study. The study team extracted bibliographic data (author, title, year, location and study design), population (age, gender distribution, occupation, comorbidities/risk factors, acute COVID-19 severity), sample size, duration of follow-up, outcome measurements, main results, post-COVID-19 symptoms and results on RTW/ work ability.

### Statistical analysis

A random effects proportional meta-analysis [41] was used to compute a pooled estimate of the RTW rate and respective 95% confidence intervals (CIs). When conducting a meta-analysis on prevalence and encountering heterogeneity in prevalence estimates across studies, it is recommended to employ a random effects model [42]. This is because a fixed effects model may yield misleading outcomes when significant heterogeneity is present, which is the case in current study. The analysis was performed using the DerSimonian and Laird random-effect [43]. The statistical heterogeneity between the studies was determined by the τ^2^ and I^2^. I^2^ indexes of 25%, 50%, and 75% indicated low, moderate, and high heterogeneity, respectively [44]. Also, the corresponding sample sizes were considered. *Events* classified the number of individuals who successfully achieved RTW, while *Total* represented the overall number of individuals, from which RTW rates could be derived. Publication bias was evaluated through a visual inspection of funnel plots. Statistical analysis was conducted using R [45] and RStudio (version 4.3.0) [46] and statistical significance was set at *p* < 0.05. The forest plot was generated using the “tidyverse” [47], “meta” [48], and “metafor” [49] packages in R. When conducting the meta-analysis, studies that did not provide data for analysing RTW rates were excluded from the analysis. This ensured that only studies with comprehensive RTW rates were included in the meta-analysis. No subgroup meta-analysis could be performed due to the to the limited number of studies providing data on RTW. A narrative, qualitative summary of the work ability of patients with post-COVID-19 was carried out and is presented in text and table form to summarise and explain the characteristics and findings of the included studies.

**Fig. 2:**
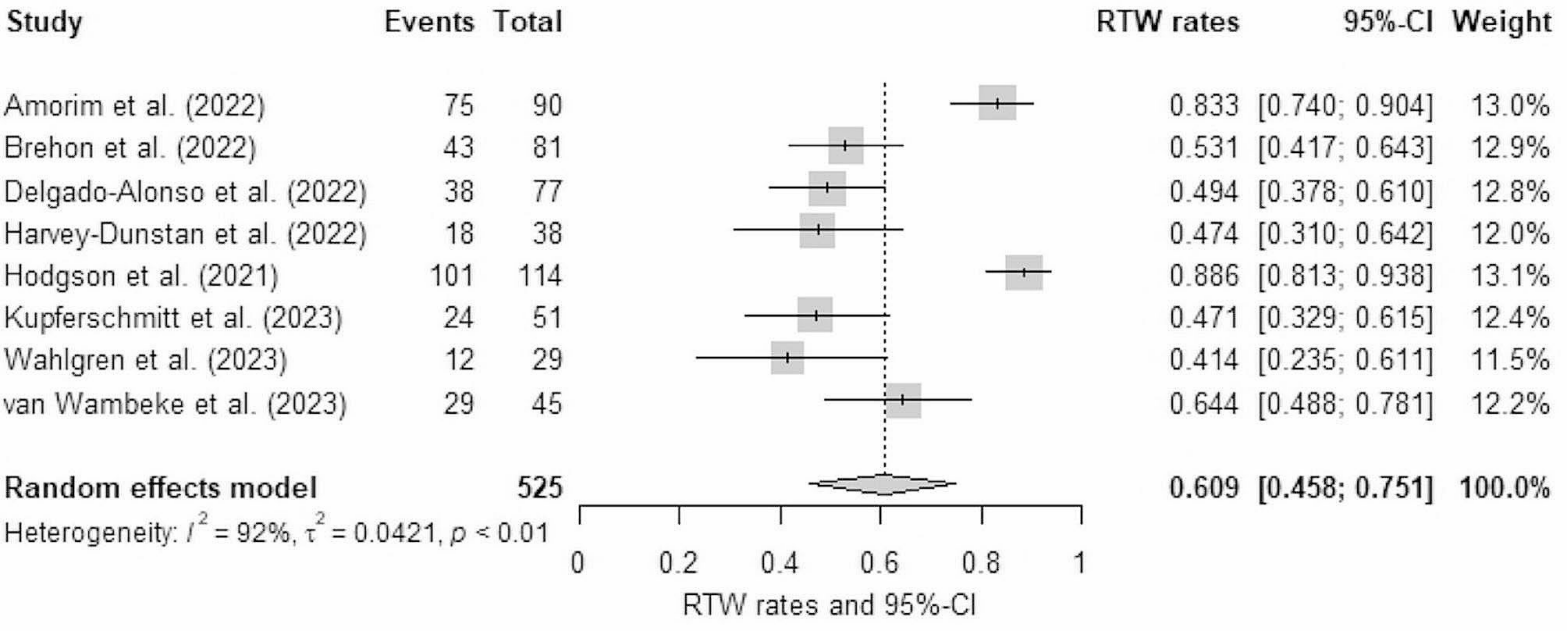
Meta-analysis of RTW of post-COVID-19 patients

**Table 1:** Impact of post-COVID-19 on work ability

Study	Sample size (*n*)	Follow-up time	Work status	Work ability	Limitations to work duties/hours
Kupferschmitt et al. 2023 [50]	51	at least 3 months	• 28/51 (54.9%) unable to work on admission • sick leave before admission: 43.1% >6 months, 13.7% 3–6 months, 41% <3months • 18/51 (35.3%) ability to work (at least 6 h/day) at discharge	N/A	• 6/51 (11.8%) gradual reintegration
Westerlind et al. 2021 [51]	11.955	4 months	• 1.592/11.955 (13.3%) sick leave for at least 12 weeks	N/A	N/A
Rutsch et al. 2023 [52]	178	5 months	• 88/178 (49%) sick leave (M: 21 weeks) • 32% restoration of their work ability after rehabilitation	• WAS (0–10) pre reha: M: 4	N/A
Kedor et al. 2022 [53]	42	6 months	N/A	• Bell disability score (0-100) Mdn.: 40 and 50 of 100 at the time of the survey	N/A
Davis et al. 2021 [25]	3.505	3–7 months	• 957/3.505 (27.3%) working same hours as before • 817/3.505 (23.3%) not working	N/A	• 1.598/3.505 (49.3%) working reduced hours
Nielsen et al. 2022 [54]	401	246 days	• 158/401 (39.4%) working same hours as before • 215/401 (53.6%) sick leave	N/A	N/A
Buonsenso et al. 2022 [55]	155	12months	• 132/154 (85.7%) patients same occupational status as before COVID-19 • 22/154 (14.3%) changed status • 7 sick leave	N/A	• 3 patients shortening of working hours
Kisiel et al. 2022 [56]	336	12 months	• 55/158 (35%) sick leave during the follow-up period (M: 8.1 weeks, SD: 6.5)	• Work ability had decreased in patients with persistent symptoms at the 12-month follow-up	N/A
Diem et al. 2022 [57]	268	13 months	• 168/268 (62.7%) inability to work • sick leave M: 26.6 weeks (95%CI 23.5-29.6)	• people with reported inability to work ◊ more commonly fatigue (*p* < 0.001), pain (*p* = 0.008), sleep disturbances (*p* = 0.030)	N/A
Müller et al. 2023 [58]	127	408.81 days	• inability to work: 86/127 (67.7%) pre reha; 90/124 (72.5%) after reha • 69.8% of the patients reported poor, 29.3% moderate, and 0.9% good work ability	• WAI (7–49): total score pre reha: Mdn.: 24.75 (IQR: 21–28); post reha: Mdn.: 24.75 (IQR: 21–28)	N/A
Peters et al. 2022 [59]	1.406	3–15 months	N/A	• WAS (0–10): before COVID-19: M: 9.3 (SD: 1.2); at the time of the survey: M:6.8 (SD: 2.2)	N/A
Sansone et al. 2022 [60]	247	15 months	N/A	• adopted WAI (1–6): Group A (symptoms < 200 days): M: 5.18 (SD: 1.08) at the time of the survey; Group B (200 + days): M: 4.5 (SD: 1.44); Group A significantly higher (*p* < 0.001)	N/A
Delgado-Alonso et al. 2022 [61]	77	20.71 months	• 38 (49.4%) patients were working • 36 (46.8%) sick leave M: 12.07, SD: 8.07 months	• not working associated with higher levels of fatigue (U = 372.50; *p* < 0.001) and lower cognitive performance (U = 488.50; *p* = 0.010)	• 12/77 (16%) reduced working hours • 18/77 (23%) job adaptation
Van Wambeke et al. 2023 [62]	45	22 months	• 18 (40%) working full-time; 16 (36%) no return-to-work	N/A	• 8 (18%) working half-time; 3 (6%) working 60-70% of the time
Wahlgren et al. 2023 [18]	94	24 months	• 4 months: 65/94 (69.1%) working; 22/94 (23.4%) sick leave • 24 months: 66/94 (70.2%) working, 15/94 (16%) sick leave	N/A	N/A

N/A = not available;  M = mean; Mdn = Median; SD = standard deviation; IQR = interquartile range; U = Mann–Whitney U test

### Risk of bias in individual studies

The risk of bias of the included cohort studies was evaluated with the Newcastle Ottawa Scale (NOS) [63], modified for cohort and cross-sectional studies [22]. The NOS’s utilizes a star system. The cohort tool assigns a maximum of 9 stars in 3 domains: (1) selection of study groups (max. 4 stars), (2) comparability (max. 2 stars), and (3) ascertainment of outcome (max. 3 stars). In the cross-sectional tool a maximum of 9 stars for quality assessment across the same 3 domains can be attained: (1) selection of study groups (max. 5 stars), (2) comparability (max. 2 stars), and (3) ascertainment of outcome (max. 2 stars). A higher score indicates a higher quality of the study. The total score could be categorized into three groups: low quality (0–4 stars), moderate quality (5–6 stars), high quality (7–9 stars). The risk of bias was assessed independently by two review authors and results were corroborated, with discrepancies resolved through discussion. Modified NOS’s (see Appendix 4) and methodological quality rankings for each study type are provided.

## Results

### Search results and study selection

The comprehensive search identified a total of 5.967 articles across the five databases (MEDLINE, EMBASE, CINAHL, CENTRAL and WHO COVID 19) and 8 additional records through other sources (see Fig. 1). After duplicates were removed, 4.625 references remained for the initial screening by title and abstract. This screening resulted in a total of 4.571 excluded articles. The remaining 54 articles were screened by full text, of which 35 did not meet the inclusion criteria with following reasons: wrong study population (*n* = 13), wrong outcome (*n* = 10), no full-text available (*n* = 6), wrong follow-up time (*n* = 2), wrong study design (*n* = 2), duplicate (*n* = 1) and wrong language (*n* = 1). 19 studies met the inclusion criteria and were included in the systematic review [18, 25, 50-62, 64-67].

### Study characteristics

The characteristics of the 19 studies included are reported in Table 2. The studies were published between 2021 and 2023. Regarding study design, 12 studies corresponded to cohort studies and 7 to cross-sectional studies. The overall population included 21.155 patients (65.3% female; 34.7% male). Study sample sizes ranged from 42 to 11.955 patients with post-COVID-19 (mean: 1.113). Most studies included middle aged participants (on average 49.15 years of age). The mean follow-up time was 11 months (range 3 months – 24 months). In all, 14 studies included patients treated in the hospital (range: 6.5 − 100%), 8 studies patients treated in intensive care units (ICU) (range: 1.2 − 28.5%) during acute SARS-CoV-2 infection, 3 studies included only non-hospitalized patients and 2 studies did not provide information. The studies were conducted in 14 different countries, of which 5 were implemented in Germany, 3 in Sweden, 2 each in Italy and the United Kingdom and 1 each in Brazil, Canada, Spain, Switzerland, Australia, Denmark and France.

**Table 2:** Characteristics of the study population

Study	Country	Study design	Sample size (*N*)	Sex (% female)	Mean (M) age (SD) or Median (Mdn) age (IQR)	Follow-up time	Acute COVID-19 severity
Amorim et al. 2022 [64]	Brazil	cohort study	780	317 (40.6%)	M: 48 (12)	3 months	352 (45.1%) hospitalized; 131 (16.8%) intensive care unit (ICU)
Brehon et al. 2022 [65]	Canada	cohort study	81	52 (64.2%)	M: 48.9 (10.5)	165.2 days	N/A
Buonsenso et al. 2022 [55]	Italy	cohort study	155	79 (50.9%)	M: 46.48 (7.3)	12 months	18 (11.6%) hospitalized
Davis et al. 2021 [25]	United Kingdom	cross-sectional study	3.762	2.961 (78.9%)	N/A	6 months	317 (8.4%) hospitalized
Delgado-Alonso et al. 2022 [61]	Spain	cross-sectional study	77	67 (87.0%)	M: 46.31 (7.97)	20.71 months	15 (19.5%) hospitalized; 3 (3.9%) ICU
Diem et al. 2022 [57]	Switzerland	cross-sectional study	309	249 (80.6%)	M: 44.6; range: 19–83 years	13.0 months	33 (10.7%) hospitalized; 14 (4.5%) ICU
Harvey-Dunstan et al. 2022 [66]	United Kingdom	cohort study	42	28 (66.7%)	M: 49 (10)	44 weeks	non-hospitalized patients
Hodgson et al. 2021 [67]	Australia	cohort study	160	63 (39.4%)	Mdn: 62 (55–71)	6 months	all patients were hospitalized
Kedor et al. 2022 [53]	Germany	cross-sectional study	42	28 (69.0%)	Mdn: 36.5; range: 22–57	6 months	3 (7.1%) hospitalized
Kisiel et al. 2022 [56]	Sweden	cross-sectional study	336	253 (75.3%)	M: 43.1 (13.4)	12 months	non-hospitalized patients
Kupferschmitt et al. 2023 [50]	Germany	cohort study	150	39 (76.5%)	M: 52.32 (9.16)	at least 3 months	N/A
Müller et al. 2023 [58]	Germany	cohort study	127	97 (76.4%)	M: 50.62 (10.74)	408.81 days	33 (25.9%) hospitalized; 10 (7.9%) ICU
Nielsen et al. 2022 [54]	Denmark	cross-sectional study	448	325 (72.5%)	M: 46.8 (12.6)	246 days	63 (14.1%) hospitalized
Peters et al. 2022 [59]	Germany	cross-sectional study	2.053	1.677 (81.7%)	Mdn: 51	3–15 months	133 (6.5%) hospitalized; 35 (1.7%) ICU
Rutsch et al. 2023 [52]	Germany	cohort study	221	145 (65.6%)	M: 52.4 (9.0)	5 months	82 (37%) hospitalized; 18 (8.1%) ICU
Sansone et al. 2022 [60]	Italy	cohort study	247	159 (64.4%)	M: 48.1 (10.5)	15 months	108 (43.7%) hospitalized, 3 (1.2%) ICU
van Wambeke et al. 2023 [62]	France	cohort study	45	28 (62.2%)	M: 49.6 (11.2)	15.1 months and 22.6 months	non-hospitalized patients
Wahlgren et al. 2023 [18]	Sweden	cohort study	165	61 (37.0%)	M: 61 (13)	24 months	100% hospitalized; 47 (28.5%) ICU
Westerlind et al. 2021 [51]	Sweden	cohort study	11.955	7.129 (59.6%)	M: 48 (11.3)	4 months	2.960 (24.8%) hospitalized

N/A = not available; M = mean; Mdn = Median; SD = standard deviation; IQR = interquartile range

### Impact of post-COVID-19 on work ability

Out of the 19 studies included in the review, 15 of them providing data on the impact of post-COVID-19 on work ability. The following analysis summarizes the key findings from each study regarding work status, sick leave, work ability, and limitations in work duties/hours and taking into account different follow-up periods (see Table 1).

#### Follow-up less than 12 months

Many post-COVID-19 patients experienced a prolonged recovery period after COVID-19, leading to temporary or long-term work limitations. Five studies had a follow-up time less than 12 months after the acute SARS-CoV-2 infection. The studies revealed that even individuals with mild or moderate acute SARS-CoV-2 infection required an extended period to recover their pre-illness work capacity. In the study by Davis et al. [25] 957 (27.3%) participants were able to maintain the same working hours as before the onset of infection. 817 (23.3%) patients were not working 3–7 months after the SARS-CoV-2 infection. Additionally, 1.598 (49.3%) participants were working reduced hours, suggesting some limitations in their work capacity. The cross-sectional study conducted by Nielsen et al. [54] with a follow-up of approximately 8 months reported a higher percentage of participants working the same hours as before the SARS-CoV-2 infection. Specifically, 39.4% of the participants in the study were able to continue working to the same extent as they did prior to the infection, while 215 out of 401 (53.6%) were on sick leave (84/215 full-time sick leave; 131/215 part-time sick leave). The national registry-based study by Westerlind et al. [51] involved 11.955 patients (follow-up: 4 months) and reported that 1.592 (13.3%) were on full-time sick leave for post-COVID-19. Kedor et al. [53] consisted a cross-sectional study of 42 post-COVID-19 patients with a follow-up of 6 months and highlighted that participants had a median Bell disability score of 40 (post-COVID/ME/CFS-group) and 50 (post-COVID/non-ME/CFS-group) indicating limited work ability (reduced working hours or inability to work). Kupferschmitt et al. [50] and Rutsch et al. [52] are two studies that evaluated work ability following rehabilitation. Kupferschmitt et al. [50] examined a sub-sample of 51 post-COVID-19 patients. Out of the 51 individuals assessed, 28 (54.9%) were unable to work on admission. At the time of discharge, 18 participants (35.3%) showed an ability to work at least 6 h per day. Additionally, 6 patients (11.8%) underwent gradual reintegration. Prior to admission, a significant proportion of participants (43.1%) were on sick leave for over 6 months, 13.7% for 3–6 months, and 41% for less than 3 months. 2.0% were not employable. In the study conducted by Rutsch et al. [52], the rehabilitation took place 5 months after SARS-CoV-2 infection. The study reported that 32% of participants experienced restoration of their work ability after rehabilitation. The mean Work Ability Score (WAS) of the Work Ability Index (WAI) was 4 on a scale of 0–10, indicating some limitations in work capacity. 41% perceive their work ability as permanently at risk. Among the participants, 88 out of 178 (49%) were on sick leave, with an average duration of 21 weeks.

#### Follow-up between 12 months and 18 months

In 15 studies that provided data on the impact of post-COVID-19 on work ability, there were 6 studies with a follow-up period between 12 months and 18 months. In the study of Buonsenso et al. [55] with a sample size of 154 participants, the majority (85.7%) maintained the same occupational status as before COVID-19. However, 22 patients (14.3%) experienced a change in their work status with following reasons: sick leave (*n* = 7), loss of job due to ill health (*n* = 3), shortening of working hours (*n* = 3), fired (*n* = 1), different reasons (*n* = 7). Kisiel et al. [56] included 158 post-COVID-19 patients followed up for 12 months. Among the 158 participants, 35% were on sick leave during the follow-up period, with an average duration of 8.1 weeks. Patients with persistent symptoms at the 12-month follow-up reported a decrease in work ability. With a follow-up of 13 months, Diem et al. [57] highlighted that 168 patients (62.7%) were unable to work, and the average sick leave duration was 26.6 weeks. There was a significant association between inability to work and symptoms such as fatigue, pain, and sleep disturbances. Müller et al. [58] performed a study on a total of 127 patients, and had a median time between SARS-CoV-2 infection and beginning of rehabilitation of 408.81 days. Among the participants, 90 (72.5%) were unable to work after rehabilitation. The majority of patients reported poor (69.8%), 29.3% moderate, and only 0.9% good work ability measured by the WAI. WAI-scores (scale 7–49) before (Median (Mdn): 24.75) and after (Mdn: 24.75) rehabilitation did not show significant changes. The study by Peters et al. [59] involved a large sample of 1.406 post-COVID-19 patients. The WAS-scores (scale 0–10) decreased from 9.3 before COVID-19 to 6.8 at the time of the survey, indicating a decline in work ability over time. The authors showed that the work ability was significant different between patients with symptoms > 3 months and patients without symptoms. Sansone et al. [60] conducted a study with 247 participants who were followed up for 15 months. The findings reveal that participants with symptoms lasting 200 or more days (Mean (M): 4.5 ± 1.44) had significantly lower mean work ability-scores (scale 1–6) compared to those with symptoms lasting less than 200 days (M: 5.18 ± 1.08; *p* < 0,001).

#### Follow-up more than 18 months

Some individuals experienced a prolonged recovery period after COVID-19, leading to long-term work limitations. Three studies had a follow-up time of 15 months and longer. Delgado-Alonso et al. [61] involved 77 participants who were followed up for an average of 20.71 months. Out of the participants, 38 (49.4%) were working, while 39 (50.6%) were not working. Among those who were currently not working, 36 (92.3%) were on sick leave. A portion of the participants (16%) reported reduced working hours, and 23% required job adaptation (e.g., more breaks, telework, cognitive aids, or a position change). Factors contributing to work disability include higher levels of fatigue, and lower cognitive performance. The cohort study by van Wambeke et al. [62] included 45 participants who were followed up for 22 months. Among these participants, 18 (40%) patients were working full-time, 3 (6%) working 60-70% of the time, 8 (18%) working half-time and the remaining individuals (36%) did not RTW. Among the mentioned studies, Wahlgren et al. [18] conducted the longest follow-up period with 24 months. At the 4-month follow-up, the majority (69.1%) of the participants were working, while a smaller proportion (23.4%) were on sick leave. At the 24-month follow-up, a similar percentage (66 out of 94 patients) were working, and a smaller proportion (16 out of 94 patients; 16%) were on sick leave.

### Impact of post-COVID-19 on return-to-work

The meta-analysis included eight studies that examined the RTW outcomes of patients previously infected with SARS-CoV-2 (Fig. 2). The random-effects meta-analysis estimated a pooled proportion of 0.609 (95% CI: 0.458–0.751), indicating that approximately 60.9% of post-COVID-19 patients were able to successfully RTW 12 or more weeks following the SARS-CoV-2 infection. In the Forest Plot, the dashed line represents the aggregated average RTW rate across all studies. Studies to the right of this line tend to indicate higher RTW rates, while those to the left suggest lower rates. Among the individual studies, Hodgson et al. [67] had the highest weight (13.1%) and reported a proportion of 0.886 (95% CI: 0.813–0.938), suggesting a high likelihood of successful RTW. The remaining studies had weights ranging from 11.5 to 13.0% and reported proportions ranging from 0.414 to 0.833. Heterogeneity analysis yielded an I^2^ index of 92% and a τ^2^ of 0,042 with *p* < 0.01, indicating substantial variability and inconsistency. Visual inspection of funnel plot asymmetry for the RTW meta-analysis did not suggest the presence of publication bias (Appendix 5), and the Peters’ regression test (intercept = 1.111; standard error (SE) = 0.147; *p* = 0.146) was not statistically significant.

### Factors influencing the work ability and return-to-work of post-COVID-19 patients

Based on the information provided from various studies, several influencing factors for work ability and RTW of post-COVID-19 patients could be identified. The duration between symptom onset and the beginning of rehabilitation or treatment influences the likelihood of returning to work [65]. Early intervention and rehabilitation improve the chances of returning to work. Job adaptations and modified duties, such as reduced working hours, tasks with lower physical or mental strain, telework or flextime can positively affect work ability and facilitate the RTW [25, 65]. Economic factors and financial needs can force post-COVID-19 patients to continue working or RTW sooner despite ongoing symptoms [25]. An individuals’ work ability can be significantly impacted by various psychological factors, among which include high levels of fatigue, depressive symptoms, and reduced cognitive performance. These factors are closely linked with diminished work capacity and overall effectiveness in the workplace [61]. In addition, Diem et al. [57] reported, that inability to work is commonly reported alongside symptoms such as fatigue, sleep disturbances, and pain. These symptoms act as significant barriers for post-COVID-19 patients, impeding their ability to engage in work-related activities and having a negative impact on overall performance and productivity. The presence of fatigue is also associated with a lower likelihood of returning to previous work hours [66]. Additionally, certain demographic and health-related factors have been associated with higher odds of not returning to work after SARS-CoV-2 infection. According to the study conducted by Westerlind et al. [51], factors such as older age, being male, having a history of sick leave before contracting COVID-19, and having received inpatient care are all associated with an increased probability of not returning to work. Overall, the influencing factors for work ability and RTW of post-COVID-19 patients are diverse and can vary between individuals, interacting in complex ways to determine work outcomes.

### Post-COVID-19 symptoms

Studies [61–66] have underscored the impact of post-COVID-19 symptoms on an individual’s work ability and RTW. Therefore, it is crucial to outline the prevalent post-COVID-19 symptoms reported in the included studies. 13 studies investigated self-reported post-COVID-19 symptoms in COVID-19 patients 12 or more weeks following diagnosis. The studies reported on a wide range of post-COVID-19 symptoms experienced by patients. Appendix 3 presents the five most commonly reported symptoms in the included studies, along with their respective prevalence rates. The prevalence of post-COVID-19 symptoms varied across studies, with estimates ranging from 12.2 to 100% of individuals who had recovered from the acute phase of the illness. Fatigue was the most commonly reported symptom, with prevalence rates exceeding 80% in many studies (mean prevalence: 72.9%). Other frequently reported symptoms included neurocognitive disorders such as concentration impairment, dizziness or memory problems. Estimates of neurocognitive symptoms prevalence ranged from 14 to 92% (mean prevalence: 59.5%). Most of the studies also reported physical ailments such as weakness, muscle pain or exercise intolerance with prevalence rates between 13% and 100% (mean prevalence: 56.2%). Other frequently reported symptoms included shortness of breath, headache, and sleep disturbances. Long-term follow-up studies indicated that patients with post-COVID-19 continued to experience symptoms for up to two years after the initial infection [18, 60–62]. For a comprehensive understanding of the full range of post-COVID-19 symptoms, it is recommended to refer to the original studies included in this systematic review.

### Risk of bias

Of the 19 studies, more than half were assessed to be of moderate quality (*n* = 10). Five studies were considered to be of high quality, and the remaining studies (*n* = 4) were considered to be of poor quality. Taken together, the NOS rating of the component studies was moderate, evidenced by mean scores of 6.2 for cohort studies and 4.8 for cross-sectional studies. The NOS quality assessment results for cohort studies are summarized in Table 3, and quality assessment results for cross-sectional studies are summarized in Table 4.

**Table 3:** Newcastle-Ottawa Quality Assessment Scale criteria for cohort studies

Study	Selection (max. ★★★)				Comparability (max. ★★)	Outcome (max. ★★★)			Total	Quality score
	Representa-tiveness of the exposed cohort	Selection of the non-exposed cohort	Ascertain-ment of exposure	Outcome not present at start of study	Comparability of cohorts on the basis of the design or analysis	Assessment of outcome	Follow-up long enough for outcomes to occur	Adequacy of follow-up of cohorts		
Amorim et al. 2022 [64]	0	0	★	★	★	★	★	★	6★	moderate
Brehon et al. 2022 [65]	★	0	★	★	★	★	★	0	6★	moderate
Buonsenso et al. 2022 [55]	★	0	0	★	★	★	★	0	5★	moderate
Harvey-Dunstan et al. 2022 [66]	0	0	★	0	★	★	★	0	4★	low
Hodgson et al. 2021 [67]	★	0	★	★	★	★	★	★	7★	high
Kupferschmitt et al. 2023 [50]	★	★	★	★	★	★	★	★	8★	high
Müller et al. 2023 [58]	★	0	★	★	★★	★	★	★	8★	high
Rutsch et al. 2023 [52]	★	0	★	0	0	★	★	★	5★	moderate
Sansone et al. 2022 [60]	★	0	★	0	★★	★	★	★	7★	high
van Wambeke et al. 2023 [62]	★	0	★	★	★	★	★	★	7★	high
Wahlgren et al. 2023 [18]	★	0	0	0	★	★	★	★	5★	moderate
Westerlind et al. 2021 [51]	★	0	★	0	★	★	★	★	6★	moderate

Quality score: high = 9 − 7 ★, moderate = 6 − 5 ★, low = 4 or fewer ★

**Table 4:** Newcastle-Ottawa Quality Assessment Scale criteria for cross-sectional studies

Study	Selection (max. ★★★)				Comparability (max. ★★)	Outcome (max. ★★★)		Total	Quality score
	Representa-tiveness of the exposed cohort	Sample size	Non-respondents	Ascertainment of the exposure (risk factor)	Comparability of subjects on the basis of design or analysis	Assessment of outcome	Statistical test		
Davis et al. 2021 [25]	★	0	0	0	0	0	★	2★	low
Delgado-Alonso et al. 2022 [61]	★	0	0	★★	★	★	★	6★	moderate
Diem et al. 2022 [57]	★	0	0	0	★★	0	★	4★	low
Kedor et al. 2022 [53]	0	0	0	★★	★★	★	★	6★	moderate
Kisiel et al. 2022 [56]	★	0	0	★★	★★	0	★	6★	moderate
Nielsen et al. 2022 [54]	0	0	0	★★	0	★	★	4★	low
Peters et al. 2022 [59]	★	0	0	★★	★	★	★	6★	moderate

Quality score: high = 9 − 7 ★, moderate = 6 − 5 ★, low = 4 or fewer ★

#### Selection

Within the cohort studies (*n* = 12), nearly all studies scoring a star for being either truly or somewhat representative of the average target population. Common methodological limitations were the failure to include a non-exposed group in cohort studies, and to ascertain whether outcomes were present prior to SARS-CoV-2 infection. The exposure (COVID-19) was usually measured using either objective measurement (e.g., polymerase chain reaction (PCR) test) or clinical judgment. Within cross-sectional studies (*n* = 7), all but two studies had somewhat representative or truly representative samples (with selection bias). However, nonresponse characteristics (with non-response/self-selection bias); and a sample size justification were not provided or poorly described in all of the cross-sectional studies. 5 out of 7 studies used validated measurement tools.

#### Comparability

Cohort studies controlled for confounders in 12 of the 13 studies. However, only two studies controlled for age, sex, and an additional factor required to score two stars. One study scored zero stars, as it used unadjusted analyses. In the cross-sectional studies, 5 studies used adjusted analyses.

#### Outcome

Within the cohort studies, all studies used a validated objective assessment tool (e.g., WAI) or a structured/systematic interview conducted by a trained healthcare/research professional and were followed up after a sufficient duration (3 months). The follow-up cohort rate was inadequate in 3 studies, as no description of differences in responders and non-responders was provided, or less than 80% responded. Within the cross-sectional studies, 3 studies used a validated objective assessment tool or a structured/systematic interview conducted by a trained healthcare/research professional; therefore, they scored a star. 3 studies scored zero stars, as they used self-reported work ability measurements. All of the cross-sectional studies were considered to have used appropriate and clearly described statistical tests.

## Discussion

The present systematic review aimed to assess the impact of post-COVID-19 on work ability and the RTW of patients previously infected with SARS-CoV-2. Through a comprehensive analysis of the available literature, we have identified several key findings that shed light on the long-term consequences of COVID-19 on individuals’ ability to work and their journey back to the workforce. The comprehensive search and rigorous study selection process resulted in the inclusion of 19 relevant studies published between 2021 and 2023, involving a diverse population of 21.155 patients from 14 different countries.

### Work ability and return-to-work

An essential determinant of a sustainable RTW is the perceived work ability, which is more independent of the patient’s specific context compared to the aspects of returning to work [68, 69]. The impact of post-COVID-19 on work ability was assessed in 15 of the 19 included studies. The findings varied depending on the follow-up period. In studies with a follow-up period of less than 12 months, it was found that many post-COVID-19 patients experienced a prolonged recovery period, resulting in temporary or long-term work limitations. A significant proportion of patients were not working or were working reduced hours (range between 13.3% and 54.9%). Even those with mild or moderate acute SARS-CoV-2 infection required extended periods to regain their pre-illness work capacity [25]. This indicates that a significant proportion of patients face challenges in resuming their work responsibilities. However, some participants were able to maintain their pre-illness work capacity. Also, in studies with a follow-up period between 12 months and 18 months, a decline in work ability over time was observed. The percentage of patients unable to work or on sick leave was relatively high in these studies (14.3 − 67.7%). Work ability scores decreased compared to pre-COVID-19 levels, indicating limitations in work capacity. For studies with a follow-up period of more than 18 months, some individuals experienced long-term work limitations. The percentage of patients working varied across studies, with a range of 40.0–70.2%. However, it is important to note that these patients may also experience a decline in work ability, resulting in reduced work productivity. Lemhöfer et al. [70] demonstrated that more than half of the post-COVID-19 patients who were able to work experienced impairments in the physical sum score of Health-Related Quality of Life (HRQoL), resulting in reduced productivity. In this context, the concept of presenteeism gains relevance.

Presenteeism involves individuals continuing to work despite being unwell, and exerting extra efforts to manage job demands, which can exacerbate health problems [71]. The estimated costs of having a sick employee could potentially be higher than the costs of their actual absence, due to lower productivity and if illnesses become worse and chronic as a result which is associated with longer periods of absence from work [30, 72]. This becomes especially notable as a substantial proportion of participants in this systematic review remained on sick leave or needed job adaptation for 15 months or longer after the SARS-CoV-2 infection.

Fatigue remained a prevalent symptom even in the long term, which could significantly impact an individual’s ability to perform daily work tasks and maintain productivity. Especially in the case of post-infectious fatigue, a symptom that affects a considerable number of post-COVID-19 patients, results concerning the long-term course of other infectious diseases (e.g., SARS virus, Q-fever, Lyme disease ) indicate the risk of chronicity [23]. Other frequently reported symptoms, such as neurocognitive disorders, physical ailments, and sleep disturbances also contributed to work limitations and challenges in returning to work [57, 61]. Delgado-Alonso et al. [61] confirmed, that there was a wide variability of influencing post-COVID-19 symptoms on work ability among the participants. Similarly, results from Pauwels et al. [34] and Sanchez-Ramirez et al. [20] indicated, that the impact on work ability and RTW for patients with long- and post-COVID-19 is complex and varies due to the different symptomatology, disease severity during the acute infection and age. Rehabilitation can play a central role in restoring the ability to work after a SARS-CoV-2 infection [73]. According to national [74, 75] and international guidelines [76], a specific post-COVID-19 rehabilitation program is recommended to contribute to the preservation and restoration of biopsychosocial health and work ability. Positive rehabilitation effects have been demonstrated for both physical and mental health in patients with post-COVID-19 [77, 78]. After regaining work ability through rehabilitation and ongoing aftercare, the process of occupational reintegration is essential.

The RTW of post-COVID-19 patients is complex and multifaceted. The meta-analysis estimated that approximately 60.9% of post-COVID-19 patients were able to successfully RTW 12 or more weeks following the SARS-CoV-2 infection. This finding highlights the importance of understanding the long-term impacts of COVID-19 on individuals’ ability to return to their pre-infection work status. However, there was substantial heterogeneity among the studies. This suggests that differences in methodologies such as study populations, follow-up durations, and other factors might have contributed to the variability in RTW outcomes observed across the studies.

Moreover, it is crucial to recognize that this prevalence figure represents just the endpoint of a much more complex process of RTW. RTW is not a straightforward, linear process. It often involves multiple stages, including gradual reintegration, adaptations, and even job changes [34]. The coexistence of comorbidities alongside post-COVID-19 can increase the complexity of the RTW process [51]. Additionally, psychosocial factors like anxiety, depression and stress can have a significant impact, leading to delays, problems, or even making it necessary to change jobs [79]. Individual differences in resilience, coping strategies and self-efficacy are key factors in how they handle the disease and navigate the RTW process [80, 81]. Some may adapt more effectively, while others may face challenges.

The findings in this systematic review reveal that environmental and organisational factors such as the availability of workplace accommodations, supportive policies, and occupational rehabilitation programs play a crucial role in facilitating successful RTW. Workplaces that offer flexible work arrangements, including modified duties, reduced working hours and remote work options were positively related with the reintegration process [25, 65]. This is particularly relevant for healthcare workers who face high work demands and workplace stress [82]. Therefore, the possibilities for adjusting workplace conditions within the company should be given more emphasis, especially to facilitate RTW for post-COVID-19 patients with extended periods of work disability. Moreover, collaboration between healthcare providers, employers, and employees was emphasized as crucial in developing personalized RTW plans tailored to individuals’ specific needs and capabilities [83]. In order to facilitate the RTW of patients with post-COVID-19, it is necessary to develop a long-term strategy [83]. Strategies for returning to work after SARS-CoV-2- infection may be similar to programs already developed for chronic diseases [84–86]. It is important to recognize that the RTW for COVID-19 survivors may be influenced by a multitude of factors, including the severity of the infection, the presence of long-term symptoms, individual resources such as resilience or self-efficacy, the socio-economic context and the nature of their occupation. Therefore, a comprehensive understanding of these factors is necessary to facilitate the successful reintegration of COVID-19 survivors into the workforce. This is in line with results by Pauwels et al. [34] indicated, that the impact on return to the workplace for patients with long-COVID and post-COVID-19 is complex and varies due to the different symptomatology. Economic aspects such as continued wage payment during illness must also be taken into consideration. The financial losses resulting from extended absences are not sustainable for some patients in the long term and can lead to psychological disorders, e.g., depression disruption of financial wellbeing up to existential fears [87]. The risk of not achieving a successful RTW increases significantly with the duration of absence. Approximately 50% of individuals are unable to RTW after a sick leave of six months [88]. In the study by Wahlgren et al. [18], it was also found that out of the 22 patients who were on sick leave four months after the SARS-CoV-2 infection, only 11 patients managed to achieve a RTW after 24 months. The process of returning to work itself can contribute significantly to the recovery from SARS-CoV-2 infection. When individuals successfully adapt the requirements of their workplace to accommodate their existing limitations, employment can serve as an effective way to improve overall performance and reduce mental stress [89, 90]. It’s important to mention that aftercare and support or self-help groups also play an important role in assisting individuals during their RTW process. They take care of the medical, emotional as well as mental and social aspects of the individuals and can create a sense of togetherness and social inclusion.

### Post-COVID-19 symptoms

The systematic review highlights that a significant proportion of individuals who have recovered from COVID-19 experience persistent symptoms and functional impairments that can impact their work ability. Identifying and presenting these symptoms not only provided a clear insight into the challenges individuals face but also contributes to developing interventions and support measures to reduce the long-term effects of COVID-19 on work ability. Neurocognitive disorders (e.g., concentration impairment or dizziness), physical ailments (e.g., weakness or exercise intolerance), shortness of breath, headache, and sleep disturbances were commonly reported symptoms among these individuals. Fatigue emerged as the most prominent symptom, with prevalence rates exceeding 80% in many studies. The occurrence of fatigue is consistent with systematic reviews on post-COVID-19 [8, 22], indicating that persistent fatigue is a common and debilitating symptom for many individuals recovering from SARS-CoV-2 infection. According to a previous study, 40% of SARS survivors experienced chronic fatigue for an average duration of 41 months following the infection [91].

Persistent symptoms can have a significant impact on the physical and psychological capacity of post-COVID-19 patients to participate in work-related activities, resulting in lower work ability and increased sick leave. Residual impairments lasting months after the acute SARS-CoV-2 infection could also explain why some of the people returning to work required modifications in their work duties or hours. This is supported by Böckermann et al. [92], who demonstrated that poor health status is linked with a higher rate of unemployment. Lemhöfer et al. [70] already revealed, that 38% of the patients were unable to work and showed impairments in physical and mental health 3–12 months after the SARS-CoV-2 infection. Especially cognitive and physical limitations, as well as existing fatigue symptoms, were associated with reduced work capacity [93, 94]. Similar associations were also demonstrated in studies included in this systematic review [57, 61].

It’s important to note that these findings are based on the available studies, and individual experiences may vary. Additionally, the long-term effects of COVID-19 on work ability are still being researched, and further studies may provide additional insights.

### Implications

The results of this systematic review and meta-analysis generate a lot of implications for healthcare providers, occupational health professionals, employers, and policymakers. It highlights the need for a comprehensive understanding of the post-COVID-19 symptoms and their impact on work ability. Healthcare professionals should be aware of the potential long-term consequences of COVID-19 and consider appropriate rehabilitation as well as aftercare and support services to help individuals RTW. Early identification of post-COVID-19 symptoms and timely interventions may significantly improve the work ability, overall well-being of patients and the successful reintegration into the workplace. Brehon et al. [65] showed, that patients with a shorter time between the onset of SARS-CoV-2 infection and admission to a rehabilitation program had a higher likelihood of RTW. It is important to note that the long-term effects of COVID-19 on work ability and the RTW process are not limited to physical symptoms alone. The systematic review highlights the significant impact of neurocognitive health challenges, such as concentration impairment, dizziness or memory problems, on individuals’ ability to work and return to the workforce. Therefore, comprehensive support systems encompassing both physical as well as neurocognitive health interventions under consideration of individual load limitations are crucial for optimizing work outcomes in post-COVID-19 patients. Rehabilitation plays a central role in restoring workability and reintegrating into the professional routine after SARS-CoV-2 infection [73]. In accordance with national [74, 75] and international guidelines [76], a specifically designed post-COVID-19 rehabilitation aims to contribute to the preservation and recovery of biopsychosocial health and work capability within the rehabilitation management of long-/post-COVID-19 patients.

Employers may need to implement flexible work arrangements, offering support services and provide reasonable accommodations for employees recovering from SARS-CoV-2 infection to facilitate their RTW and foster a more supportive and inclusive work environment. The possibilities of adapting working conditions within the company should be given more attention, especially to enable an optimal reintegration into the profession and achieve a RTW for post-COVID-19 patients with prolonged work disability periods [65, 83]. Employers should also be educated about the potential challenges faced by post-COVID-19 patients and the importance of providing appropriate resources and support to aid in their recovery and successful RTW. Policy initiatives could focus on ensuring that post-COVID-19 patients have access to necessary healthcare and rehabilitation services, and protections against discrimination in the workplace due to COVID-19 related symptoms. Furthermore, continuous monitoring of the potential increase in the number of people opting for early retirement due to post-COVID-19 is crucial in the upcoming years, as reduced work ability serves as a predictor for such decisions [29].

### Limitations

While the systematic review provides valuable insights into the impact of post-COVID-19 on work ability and the RTW process, there are some limitations to consider. The available literature is still evolving, and the studies included in this review vary in their methodologies, leading to heterogeneity, and thus, difficulties of comparison. Additionally, there is a scarcity of long-term follow-up studies, making it challenging to ascertain the impact of post-COVID-19 on work ability and RTW. Future research should focus on longer follow-up periods to better understand how post-COVID-19 symptoms change over time and affect people’s ability to work. Large and long-term cohort studies incorporating mixed methods, encompassing both qualitative as well as quantitative approaches, are essential for gaining comprehensive insights into the long-term consequences of COVID-19. These studies allow a more differentiated understanding of the multifaceted impacts of the disease on individuals’ work ability and RTW and should aim for diverse representation in terms of age, gender, severity of acute COVID, occupation, and geographical locations. Supplementing quantitative approaches with qualitative research can offer a better understanding of the lived experiences and challenges faced by individuals recovering from post-COVID-19. Previous research has descriptively analysed work ability and RTW as a secondary parameter over mostly short periods of time. No articles were found that analysed the impact of post-COVID-19 on work ability and RTW as their primary objective. Data about work ability and RTW was collected from different questionnaires (Bell Disability Scale, World Health Organization Disability Assessment Schedule, Work ability index), open questions, online surveys or work ability scales. The studies primarily relied on self-reported work ability, which may introduce biases and problems such social desirability. Moreover, there were no articles found describing a validated screening tool for post-COVID-19. Employing standardized assessment tools for work ability and RTW outcomes will enhance the reliability and comparability of findings across studies.

There is heterogeneity across the studies with respect to the selection of participants, the assessment of outcomes, follow-up periods and sample sizes in almost all the studies which may influence the generalizability of the results of this study. Despite these limitations, the authors maintain that this systematic review significantly addresses the knowledge gap regarding the impact of post-COVID-19 on work ability and RTW.

The risk of bias assessment indicated that more than half of the included studies were of moderate quality. Common limitations included the failure to include a non-exposed group in cohort studies and inadequate control for confounders. In cross-sectional studies, nonresponse characteristics and sample size justifications were often poorly described. The Quality Assessment of the included studies using the Newcastle-Ottawa Scale has revealed certain consistency problems and its reliability relies on the expertise of the operator [95, 96]. Consequently, if conducted by a different research group, the quality assessment might have yielded varying results.

Another notable constraint of this systematic review is the restriction to articles published only in English or German. This might result in the exclusion of relevant studies published in other languages, limiting the reviews’ overall comprehensiveness.

The majority of studies included hospitalized patients, potentially impacting the generalizability of our findings to the broader population of individuals with post-COVID-19. This focus might introduce bias due to the higher prevalence of comorbidities among hospitalized patients [97]. However, previous studies demonstrated that individuals, even with mild SARS-CoV-2 infections, developed post-COVID-19 symptoms [98, 99], underscoring the importance of considering varying disease severities. To improve our understanding, future studies should include more non-hospitalized individuals, providing a more balanced perspective on the post-COVID-19 landscape.

In addition to the previously mentioned limitations, one crucial aspect that most of the included studies overlooked is the consideration of the specific COVID-19 variants by which the patients were infected. This oversight prevents us from comprehensively understanding the potential differential effects of various variants on individuals’ ability to RTW after recovering from SARS-CoV-2 infection. Notably, most studies with a large sample size were conducted in the early stages of the pandemic when the alpha and delta variants were predominant. However, the study by Aben et al. [35] provided the insight that later virus variants (e.g., Omicron) demonstrated a shorter duration between infection and RTW. This crucial finding does not receive adequate attention within the context of this systematic review.

### Unanswered questions

In addition to detailing a number of methodological considerations, this review has also highlighted gaps in the literature. The following unanswered questions will help focus future research:

- How does the individual recovery of employees’ ability to work with post-COVID-19 look like?
- How do processes of occupational reintegration post-COVID-19 employees look like, and which (promoting and hindering) factors are relevant?
- What problems arise during the recovery process to the ability to work, and how do employees deal with such problems? What coping strategies do they develop and apply?
- What influence do physical and psychological resources have on the ability to work of employees with post-COVID-19?
- What role does the social as well as the workplace environment (considering the broader context of the International Classification of Functioning, Disability, and Health) play in the recovery of employees’ ability to work?
- How do demographic factors impact the ability to work of individuals with post-COVID-19, and what tailored interventions can be developed to address specific needs based on demographic diversity?
- What are the different subtypes of post-COVID-19, and how can a deeper understanding of the various clinical manifestations post-COVID-19 enhance the effectiveness of RTW interventions?
- To what extent do specific interventions such as rehabilitation programmes, aftercare and support services for employees with post-COVID-19 result in improvements of ability to work and faster RTW?

## Conclusions

This systematic review and meta-analysis provide valuable insights into the impact of post-COVID-19 on work ability and the RTW. The findings underscore the need for comprehensive support for individuals recovering from SARS-CoV-2 infection to improve their work capacity and overall quality of life. However, the influence of post-COVID-19 on the working-age population seems to be substantial, and it is expected to result in enduring strains on economic and healthcare systems. Policymakers, healthcare providers, occupational health professionals and employers need to collaborate to create inclusive work environments and implement tailored rehabilitation and aftercare programs that improve the work ability of post-COVID-19 patients in long-term. Future research should focus on long-term follow-up studies with mixed methods (qualitative and quantitative) to gain a more comprehensive understanding of the trajectory of post-COVID-19 symptoms and their impact on work outcomes and to identify effective interventions to facilitate the RTW for affected individuals.

### Electronic supplementary material

Below is the link to the electronic supplementary material.

Supplementary Material 1

## Data Availability

All data generated or analysed during this study are included in this published article and its supplementary information files.

## Abbreviations

6MWD: Six-minute walking distance 6MWD
CI: Confidence intervals
COVID-19: Coronavirus disease 2019
DRKS: German Register for Clinical Studies
HRQoL: Health-Related Quality of Life
ICTRP: International Clinical Trials Registry Platform
ICU: Intensive care units
IQR: Interquartile range
PRISMA: Preferred Reporting Items for Systematic Reviews and Meta-Analyses
PROSPERO: International Prospective Register for Systematic Reviews
RTW: Return-to-work
PCR: Polymerase chain reaction
M: Mean
Mdn: Median
MeSH: Medical Subject Headings
NICE: National Institute for Health and Care Excellence
NOS: Newcastle Ottawa Scale
SD: Standard deviation
SE: Standard error
WAI: Work ability index
WAS: Work ability score

## References

[1] Khalatbari-Soltani S, Cumming RC, Delpierre C, Kelly-Irving M (2020). Importance of collecting data on socioeconomic determinants from the early stage of the COVID-19 outbreak onwards. J Epidemiol Community Health.

[2] Burdorf A, Porru F, Rugulies R (2021). The COVID-19 pandemic: one year later - an occupational perspective. Scand J Work Environ Health.

[3] Reuter M, Rigó M, Formazin M, Liebers F, Latza U, Castell S (2022). Occupation and SARS-CoV-2 infection risk among 108 960 workers during the first pandemic wave in Germany. Scand J Work Environ Health.

[4] Alshamrani MM, El-Saed A, Al Zunitan M, Almulhem R, Almohrij S (2021). Risk of COVID-19 morbidity and mortality among healthcare workers working in a large Tertiary Care Hospital. Int J Infect Dis.

[5] Wachtler B, Neuhauser H, Haller S, Grabka MM, Zinn S, Schaade L (2021). The risk of infection with SARS-CoV-2 among Healthcare workers during the pandemic. Dtsch Arztebl Int.

[6] Ferland L, Carvalho C, Gomes Dias J, Lamb F, Adlhoch C, Suetens C (2022). Risk of hospitalization and death for healthcare workers with COVID-19 in nine European countries, January 2020-January 2021. J Hosp Infect.

[7] Deutsche Gesetzliche Unfallversicherung (DGUV). Berufskrankheiten und Arbeitsunfälle im Zusammenhang mit COVID-19. 2023. https://www.dguv.de/medien/inhalt/mediencenter/hintergrund/covid/dguv_zahlen_covid.pdf. Accessed: 15.11.2023.

[8] Alkodaymi MS, Omrani OA, Fawzy NA, Shaar BA, Almamlouk R, Riaz M (2022). Prevalence of post-acute COVID-19 syndrome symptoms at different follow-up periods: a systematic review and meta-analysis. Clin Microbiol Infect.

[9] Almas T, Malik J, Alsubai AK, Jawad Zaidi SM, Iqbal R, Khan K (2022). Post-acute COVID-19 syndrome and its prolonged effects: an updated systematic review. Ann Med Surg (Lond).

[10] Yuan N, Lv ZH, Sun CR, Wen YY, Tao TY, Qian D (2023). Post-acute COVID-19 symptom risk in hospitalized and non-hospitalized COVID-19 survivors: a systematic review and meta-analysis. Front Public Health.

[11] Sivan M, Taylor S. NICE guideline on long covid. Br Med J Publishing Group. 2020;371. 10.1136/bmj.m4938 10.1136/bmj.m4938 33361141

[12] Wulf Hanson S, Abbafati C, Aerts JG, Al-Aly Z, Ashbaugh C, Ballouz T (2022). Estimated global proportions of individuals with persistent fatigue, cognitive, and respiratory symptom clusters following symptomatic COVID-19 in 2020 and 2021. JAMA.

[13] Thompson EJ, Williams DM, Walker AJ, Mitchell RE, Niedzwiedz CL, Yang TC (2022). Long COVID burden and risk factors in 10 UK longitudinal studies and electronic health records. Nat Commun.

[14] Peter RS, Nieters A, Kräusslich HG, Brockmann SO, Göpel S, Kindle G (2022). Post-acute sequelae of covid-19 six to 12 months after infection: population based study. BMJ.

[15] Kokolevich ZM, Crowe M, Mendez D, Biros E, Reznik JE. Most common long COVID physical symptoms in working age adults who experienced mild COVID-19 infection: a scoping review. Healthc [Internet]. 2022;10(12). 10.3390/healthcare10122577 10.3390/healthcare10122577 PMC9778298 36554098

[16] Chen C, Haupert SR, Zimmermann L, Shi X, Fritsche LG, Mukherjee B (2022). Global prevalence of Post-coronavirus Disease 2019 (COVID-19) Condition or Long COVID: a Meta-analysis and systematic review. J Infect Dis.

[17] O’Mahoney LL, Routen A, Gillies C, Ekezie W, Welford A, Zhang A, et al. The prevalence and long-term health effects of long covid among hospitalised and non-hospitalised populations: a systematic review and meta-analysis. eClinicalMedicine. 2023;55. 10.1016/j.eclinm.2022.101762 10.1016/j.eclinm.2022.101762 PMC9714474 36474804

[18] Wahlgren C, Forsberg G, Divanoglou A, Östholm Balkhed Å, Niward K, Berg S, et al. Two-year follow-up of patients with post-COVID-19 condition in Sweden: a prospective cohort study. Lancet Reg Health Eur. 2023;100595. 10.1016/j.lanepe.2023.100595 10.1016/j.lanepe.2023.100595 PMC9951394 36855599

[19] Liao T, Meng D, Xiong L, Wu S, Yang L, Wang S (2022). Long-Term effects of COVID-19 on Health Care workers 1-Year Post-discharge in Wuhan. Infect Dis Ther.

[20] Sanchez-Ramirez DC, Normand K, Zhaoyun Y, Torres-Castro R. Long-term impact of COVID-19: a systematic review of the literature and Meta-analysis. Biomedicines. 2021;9(8). 10.3390/biomedicines9080900 10.3390/biomedicines9080900 PMC8389585 34440104

[21] Morioka S, Tsuzuki S, Maruki T, Terada M, Miyazato Y, Kutsuna S (2023). Epidemiology of post-COVID conditions beyond 1 year: a cross-sectional study. Public Health.

[22] Ceban F, Ling S, Lui LMW, Lee Y, Gill H, Teopiz KM (2022). Fatigue and cognitive impairment in Post-COVID-19 syndrome: a systematic review and meta-analysis. Brain Behav Immun.

[23] Islam MF, Cotler J, Jason LA (2020). Post-viral fatigue and COVID-19: lessons from past epidemics. Fatigue: Biomed Health Behav.

[24] Gualano MR, Rossi MF, Borrelli I, Santoro PE, Amantea C, Daniele A (2022). Returning to work and the impact of post COVID-19 condition: a systematic review. Work.

[25] Davis HE, Assaf GS, McCorkell L, Wei H, Low RJ, Re’em Y (2021). Characterizing long COVID in an international cohort: 7 months of symptoms and their impact. EClinicalMedicine.

[26] Ilmarinen J, Tempel J (2002). Arbeitsfähigkeit 2010 - was können wir tun, damit sie gesund bleiben?.

[27] Ilmarinen J. Towards a longer worklife! Ageing and the quality of Worklife in the European Union. Z für Arbeits- und Organisationspsychologie A&O. 2006;52. 10.1026/0932-4089.52.1.47

[28] Slebus FG, Kuijer PP, Willems JH, Sluiter JK, Frings-Dresen MH (2007). Prognostic factors for work ability in sicklisted employees with chronic diseases. Occup Environ Med.

[29] Lundin A, Kjellberg K, Leijon O, Punnett L, Hemmingsson T (2016). The Association between Self-assessed future work ability and long-term sickness absence, disability pension and unemployment in a General Working Population: a 7-Year Follow-Up study. J Occup Rehabil.

[30] Gandjour A, Long COVID (2023). Costs for the German economy and health care and pension system. BMC Health Serv Res.

[31] Rashid M, Heiden M, Nilsson A, Kristofferzon ML (2021). Do work ability and life satisfaction matter for return to work? Predictive ability of the work ability index and life satisfaction questionnaire among women with long-term musculoskeletal pain. BMC Public Health.

[32] Sviridova O, Michaelson P (2018). Predictors for return to work after multimodal rehabilitation in persons with persistent musculoskeletal pain. Edorium J Disabil Rehabilitation.

[33] Cancelliere C, Donovan J, Stochkendahl MJ, Biscardi M, Ammendolia C, Myburgh C (2016). Factors affecting return to work after injury or illness: best evidence synthesis of systematic reviews. Chiropr Man Th.

[34] Pauwels S, Boets I, Polli A, Mylle G, De Raeve H, Godderis L. Return to work after long COVID: Evidence at 8th March 2021. 2021. https://www.hse.gov.uk/research/assets/docs/return-to-work-after-long-covid.pdf. Accessed: 16.11.2023.

[35] Aben B, Kok RN, de Wind A (2023). Return-to-work rates and predictors of absence duration after COVID-19 over the course of the pandemic. Scand J Work Environ Health.

[36] Kamdar BB, Suri R, Suchyta MR, Digrande KF, Sherwood KD, Colantuoni E (2020). Return to work after critical illness: a systematic review and meta-analysis. Thorax.

[37] Page MJ, McKenzie JE, Bossuyt PM, Boutron I, Hoffmann TC, Mulrow CD (2021). The PRISMA 2020 statement: an updated guideline for reporting systematic reviews. BMJ.

[38] Scottish Intercollegiate Guidelines Network (SIGN). Search filters. 2021. https://www.sign.ac.uk/what-we-do/methodology/search-filters/. Accessed: 15.11.2023.

[39] Lefebvre C, Glanville J, Briscoe S, Featherstone R, Littlewood A, Metzendorf M-I et al. Chapter 4: Searching for and selecting studies. In: Higgins JPT, Thomas J, Chandler J, Cumpston M, Li T, Page MJ, editorsOctober. Cochrane Handbook for Systematic Reviews of Interventions version 6.4 (updated 2023). Cochrane, 2023.

[40] Ouzzani M, Hammady H, Fedorowicz Z, Elmagarmid A (2016). Rayyan—a web and mobile app for systematic reviews. Syst Reviews.

[41] Barker TH, Migliavaca CB, Stein C, Colpani V, Falavigna M, Aromataris E (2021). Conducting proportional meta-analysis in different types of systematic reviews: a guide for synthesisers of evidence. BMC Med Res Methodol.

[42] Wang K-S, Liu X. Statistical methods in the meta-analysis of prevalence of human diseases. J Biostatistics Epidemiol. 2016;2(1). doi.

[43] DerSimonian R, Laird N (1986). Meta-analysis in clinical trials. Control Clin Trials.

[44] Higgins JP, Thompson SG, Deeks JJ, Altman DG (2003). Measuring inconsistency in meta-analyses. BMJ.

[45] R Core Team. R: A Language and Environment for Statistical Computing. 2023. https://www.r-project.org/. Accessed: 21.11.2023.

[46] Posit team. RStudio: Integrated Development Environment for R. 2023. http://www.posit.co/. Accessed: 21.11.2023.

[47] Wickham H, Averick M, Bryan J, Chang W, McGowan L, François R, et al. Welcome to the Tidyverse. J Open Source Softw. 2019;4(43). 10.21105/joss.01686

[48] Balduzzi S, Rücker G, Schwarzer G (2019). How to perform a meta-analysis with R: a practical tutorial. Evid Based Ment Health.

[49] Viechtbauer W (2010). Conducting Meta-analyses in R with the metafor Package. J Stat Softw.

[50] Kupferschmitt A, Langheim E, Tüter H, Etzrodt F, Loew TH, Köllner V (2023). First results from post-COVID inpatient rehabilitation. Front Rehabil Sci.

[51] Westerlind E, Palstam A, Sunnerhagen KS, Persson HC (2021). Patterns and predictors of sick leave after Covid-19 and long Covid in a national Swedish cohort. BMC Public Health.

[52] Rutsch M, Frommhold J, Buhr-Schinner H, Gross T, Schüller PO, Deck R. Pneumologische Rehabilitation bei Long Covid – Gesundheitliche Veränderungen am Ende der stationären Rehabilitationsmaßnahme. Rehabilitation (Stuttg). 2023(EFirst). 10.1055/a-1964-7401 10.1055/a-1964-7401 36649730

[53] Kedor C, Freitag H, Meyer-Arndt L, Wittke K, Hanitsch LG, Zoller T (2022). A prospective observational study of post-COVID-19 chronic fatigue syndrome following the first pandemic wave in Germany and biomarkers associated with symptom severity. Nat Commun.

[54] Nielsen TB, Leth S, Pedersen M, Harbo HD, Nielsen CV, Laursen CH, et al. Mental fatigue, activities of Daily Living, Sick Leave and Functional Status among patients with long COVID: a cross-sectional study. Int J Environ Res Public Health. 2022;19(22). 10.3390/ijerph192214739 10.3390/ijerph192214739 PMC9690484 36429458

[55] Buonsenso D, Gualano MR, Rossi MF, Valz Gris A, Sisti LG, Borrelli I, et al. Post-acute COVID-19 sequelae in a Working Population at one year Follow-Up: a wide range of impacts from an Italian sample. Int J Environ Res Public Health. 2022;19(17). 10.3390/ijerph191711093 10.3390/ijerph191711093 PMC9518581 36078808

[56] Kisiel MA, Janols H, Nordqvist T, Bergquist J, Hagfeldt S, Malinovschi A, et al. Predictors of post-COVID-19 and the impact of persistent symptoms in non-hospitalized patients 12 months after COVID-19, with a focus on work ability. Ups J Med Sci. 2022;127. 10.48101/ujms.v127.8794 10.48101/ujms.v127.8794 PMC9383047 35991464

[57] Diem L, Schwarzwald A, Friedli C, Hammer H, Gomes-Fregolente L, Warncke J (2022). Multidimensional phenotyping of the post-COVID-19 syndrome: a Swiss survey study. CNS Neurosci Ther.

[58] Müller K, Poppele I, Ottiger M, Zwingmann K, Berger I, Thomas A, et al. Impact of Rehabilitation on Physical and Neuropsychological Health of patients who Acquired COVID-19 in the Workplace. Int J Environ Res Public Health. 2023;20(2). 10.3390/ijerph20021468 10.3390/ijerph20021468 PMC9864141 36674222

[59] Peters C, Dulon M, Westermann C, Kozak A, Nienhaus A. Long-Term effects of COVID-19 on Workers in Health and Social Services in Germany. Int J Environ Res Public Health. 2022;19(12). 10.3390/ijerph19126983 10.3390/ijerph19126983 PMC9222999 35742231

[60] Sansone D, Tassinari A, Valentinotti R, Kontogiannis D, Ronchese F, Centonze S, et al. Persistence of symptoms 15 months since COVID-19 diagnosis: prevalence, risk factors and residual work ability. Life. 2023;13(1). 10.3390/life13010097 10.3390/life13010097 PMC9862952 36676046

[61] Delgado-Alonso C, Cuevas C, Oliver-Mas S, Diez-Cirarda M, Delgado-Alvarez A, Gil-Moreno MJ, et al. Fatigue and cognitive dysfunction are Associated with Occupational Status in Post-COVID Syndrome. Int J Environ Res Public Health. 2022;19(20). 10.3390/ijerph192013368 10.3390/ijerph192013368 PMC9603617 36293950

[62] Van Wambeke E, Bezler C, Kasprowicz AM, Charles AL, Andres E, Geny B. Two-years Follow-Up of symptoms and return to work in Complex Post-COVID-19 patients. J Clin Med. 2023;12(3). 10.3390/jcm12030741 10.3390/jcm12030741 PMC9917586 36769389

[63] Wells G, Shea B, O’Connell J. The Newcastle-Ottawa Scale (NOS) for Assessing The Quality of Nonrandomised Studies in Meta-analyses. 2014. https://www.ohri.ca/programs/clinical_epidemiology/oxford.asp. Accessed: 16.11.2023.

[64] Amorim CEN, Gomes VLT, Cristelli MP, Viana LA, de Luca Correa H, Lima GBB (2022). High prevalence of Long-COVID among kidney transplant recipients: a longitudinal cohort study. Transplantation.

[65] Brehon K, Niemelainen R, Hall M, Bostick GP, Brown CA, Wieler M (2022). Return-to-work following Occupational Rehabilitation for Long COVID: descriptive cohort study. JMIR Rehabil Assist Technol.

[66] Harvey-Dunstan TC, Jenkins AR, Gupta A, Hall IP, Bolton CE (2022). Patient-related outcomes in patients referred to a respiratory clinic with persisting symptoms following non-hospitalised COVID-19. Chron Respir Dis.

[67] Hodgson CL, Higgins AM, Bailey MJ, Mather AM, Beach L, Bellomo R (2021). The impact of COVID-19 critical illness on new disability, functional outcomes and return to work at 6 months: a prospective cohort study. Crit Care.

[68] Gragnano A, Negrini A, Miglioretti M, Corbière M (2018). Common psychosocial factors Predicting Return to Work after Common Mental disorders, Cardiovascular diseases, and cancers: a review of Reviews supporting a Cross-disease Approach. J Occup Rehabil.

[69] Lamore K, Dubois T, Rothe U, Leonardi M, Girard I, Manuwald U, et al. Return to work interventions for Cancer survivors: a systematic review and a Methodological Critique. Int J Environ Res Public Health. 2019;16(8). 10.3390/ijerph16081343 10.3390/ijerph16081343 PMC6518012 31014004

[70] Lemhöfer C, Sturm C, Loudovici-Krug D, Guntenbrunner C, Bülow M, Reuken P, et al. Quality of life and ability to work of patients with Post-COVID syndrome in relation to the number of existing symptoms and the duration since infection up to 12 months: a cross-sectional study. Qual Life Res. 2023;1–12. 10.1007/s11136-023-03369-2 10.1007/s11136-023-03369-2 PMC9984128 36869248

[71] Kinman G (2019). Sickness presenteeism at work: prevalence, costs and management. Br Med Bull.

[72] Kigozi J, Jowett S, Lewis M, Barton P, Coast J (2017). The estimation and inclusion of Presenteeism costs in Applied Economic evaluation: a systematic review. Value Health.

[73] Asaba E, Sy M, Pineda RC, Aldrich R, Anzai T, Bontje P, et al. Return to work after COVID-19: an international perspective. World Federation Occup Therapists Bull. 2022;1–11. 10.1080/14473828.2022.2045819

[74] Koczulla AR, Ankermann T, Behrends U, Berlit P, Böing S, Brinkmann F et al. S1-Leitlinie „Post-COVID/Long-COVID. 2022. https://register.awmf.org/assets/guidelines/020-027l_S1_Post_COVID_Long_COVID_2022-08.pdf. Accessed: 01.11.2023. 10.1055/a-1551-9734 34474488

[75] Kluge S, Rabe KF. S3-Leitlinie Empfehlungen zur stationären Therapie von Patienten mit COVID-19 – Living Guideline. 2022. https://register.awmf.org/assets/guidelines/113-001LGl_S3_Empfehlungen-zur-stationaeren-Therapie-von-Patienten-mit-COVID-19_2022-09_1.pdf. Accessed: 01.11.2023.

[76] WHO. Clinical management of COVID-19: living guideline, 13 January 2023. 2023. https://www.who.int/publications/i/item/WHO-2019-nCoV-clinical-2023.1. Accessed: 01.11.2023.

[77] Bailly M, Pélissier L, Coudeyre E, Evrard B, Bingula R, Rochette C, et al. Systematic review of COVID-19-Related physical activity-based rehabilitations: benefits to be confirmed by more robust methodological approaches. Int J Environ Res Public Health. 2022;19(15). 10.3390/ijerph19159025 10.3390/ijerph19159025 PMC9331032 35897400

[78] Nopp S, Moik F, Klok FA, Gattinger D, Petrovic M, Vonbank K (2022). Outpatient Pulmonary Rehabilitation in patients with long COVID improves Exercise Capacity, Functional Status, Dyspnea, fatigue, and Quality of Life. Respiration.

[79] Stevelink SAM, Mark KM, Fear NT, Hotopf M, Chalder T (2022). Chronic fatigue syndrome and occupational status: a retrospective longitudinal study. Occup Med (Lond).

[80] Macía P, Barranco M, Gorbeña S, Álvarez-Fuentes E, Iraurgi I (2021). Resilience and coping strategies in relation to mental health outcomes in people with cancer. PLoS ONE.

[81] Aarestad SH, Harris A, Einarsen SV, Gjengedal RGH, Osnes K, Hannisdal M (2021). Exposure to bullying behaviours, resilience, and return to work self-efficacy in patients on or at risk of sick leave. Ind Health.

[82] Young KP, Kolcz DL, O’Sullivan DM, Ferrand J, Fried J, Robinson K (2021). Health Care Workers’ Mental Health and Quality of Life during COVID-19: results from a mid-pandemic, National Survey. Psychiatr Serv.

[83] Lunt J, Hemming S, Burton K, Elander J, Baraniak A (2022). What workers can tell us about post-COVID workability. Occup Med (Lond).

[84] Vooijs M, Leensen MC, Hoving JL, Wind H, Frings-Dresen MH (2015). Interventions to enhance work participation of workers with a chronic disease: a systematic review of reviews. Occup Environ Med.

[85] Wegrzynek PA, Wainwright E, Ravalier J (2020). Return to work interventions for chronic pain: a systematic review. Occup Med (Lond).

[86] Popa AE, Bejenaru A, Mitrea EC, Morandau F, Pogan L. Return to work after chronic disease: a theoretical framework for understanding the worker-employer dynamic. Chronic Illn. 2022;17423953221117852. 10.1177/17423953221117852 10.1177/17423953221117852 35912437

[87] Wong J, Kudla A, Pham T, Ezeife N, Crown D, Capraro P (2021). Lessons learned by Rehabilitation Counselors and Physicians in Services to COVID-19 Long-Haulers: a qualitative study. Rehabilitation Couns Bull.

[88] Blank L, Peters J, Pickvance S, Wilford J, Macdonald E (2008). A systematic review of the factors which predict return to work for people suffering episodes of poor mental health. J Occup Rehabil.

[89] Karanika-Murray M, Biron C (2019). The health-performance framework of presenteeism: towards understanding an adaptive behaviour. Hum Relat.

[90] Tan W, Hao F, McIntyre RS, Jiang L, Jiang X, Zhang L (2020). Is returning to work during the COVID-19 pandemic stressful? A study on immediate mental health status and psychoneuroimmunity prevention measures of Chinese workforce. Brain Behav Immun.

[91] Lam MH, Wing YK, Yu MW, Leung CM, Ma RC, Kong AP (2009). Mental morbidities and chronic fatigue in severe acute respiratory syndrome survivors: long-term follow-up. Arch Intern Med.

[92] Böckerman P, Ilmakunnas P (2009). Unemployment and self-assessed health: evidence from panel data. Health Econ.

[93] Haller J, Kocalevent RD, Nienhaus A, Peters C, Bergelt C, Koch-Gromus U (2022). Persistent fatigue symptoms following COVID-19 infection in healthcare workers: risk factors and impact on quality of life. Bundesgesundheitsblatt Gesundheitsforschung Gesundheitsschutz.

[94] Hasenoehrl T, Palma S, Huber DFX, Kastl S, Steiner M, Jordakieva G, et al. Post-COVID: effects of physical exercise on functional status and work ability in health care personnel. Disabil Rehabil. 2022;1–7. 10.1080/09638288.2022.2111467 10.1080/09638288.2022.2111467 35980383

[95] Hartling L, Hamm M, Milne A, Vandermeer B, Santaguida PL, Ansari M (2012). Validity and Inter-rater Reliability Testing of Quality Assessment Instruments.

[96] Lo CK, Mertz D, Loeb M (2014). Newcastle-Ottawa Scale: comparing reviewers’ to authors’ assessments. BMC Med Res Methodol.

[97] Pérez-González A, Araújo-Ameijeiras A, Fernández-Villar A, Crespo M, Poveda E (2022). Long COVID in hospitalized and non-hospitalized patients in a large cohort in Northwest Spain, a prospective cohort study. Sci Rep.

[98] Reme BA, Gjesvik J, Magnusson K (2023). Predictors of the post-COVID condition following mild SARS-CoV-2 infection. Nat Commun.

[99] Lund LC, Hallas J, Nielsen H, Koch A, Mogensen SH, Brun NC (2021). Post-acute effects of SARS-CoV-2 infection in individuals not requiring hospital admission: a Danish population-based cohort study. Lancet Infect Dis.

