# Supplementary material for: Work ability and return-to-work of patients with post-COVID-19: a systematic review and meta-analysis
Source: BMC Public Health. 2024 Jul 7;24:1811. doi: 10.1186/s12889-024-19328-6 (PMC11229229; doi:10.1186/s12889-024-19328-6)
Supplement: Supplementary file 1 — Supplementary Material 1 [file 12889_2024_19328_MOESM1_ESM.docx]

**Appendix 1**

**Table S1** PRISMA Statement

| **Section and Topic** | **Item** | **Checklist item** | **Location where item is reported** |
| --- | --- | --- | --- |
| **TITLE** | | |  |
| Title | 1 | Identify the report as a systematic review. | p.1 |
| **ABSTRACT** | | |  |
| Abstract | 2 | See the PRISMA 2020 for Abstracts checklist. | p.2 |
| **INTRODUCTION** | | |  |
| Rationale | 3 | Describe the rationale for the review in the context of existing knowledge. | p.4-6 |
| Objectives | 4 | Provide an explicit statement of the objective(s) or question(s) the review addresses. | p.6 |
| **METHODS** | | |  |
| Eligibility criteria | 5 | Specify the inclusion and exclusion criteria for the review and how studies were grouped for the syntheses. | p.7-8 |
| Information sources | 6 | Specify all databases, registers, websites, organisations, reference lists and other sources searched or consulted to identify studies. Specify the date when each source was last searched or consulted. | p.6-7 |
| Search strategy | 7 | Present the full search strategies for all databases, registers and websites, including any filters and limits used. | Appendix 2 |
| Selection process | 8 | Specify the methods used to decide whether a study met the inclusion criteria of the review, including how many reviewers screened each record and each report retrieved, whether they worked independently, and if applicable, details of automation tools used in the process. | p.8 |
| Data collection process | 9 | Specify the methods used to collect data from reports, including how many reviewers collected data from each report, whether they worked independently, any processes for obtaining or confirming data from study investigators, and if applicable, details of automation tools used in the process. | p.8 |
| Data items | 10a | List and define all outcomes for which data were sought. Specify whether all results that were compatible with each outcome domain in each study were sought (e.g. for all measures, time points, analyses), and if not, the methods used to decide which results to collect. | p.8 |
|  | 10b | List and define all other variables for which data were sought (e.g. participant and intervention characteristics, funding sources). Describe any assumptions made about any missing or unclear information. | p.8 |
| Study risk of bias assessment | 11 | Specify the methods used to assess risk of bias in the included studies, including details of the tool(s) used, how many reviewers assessed each study and whether they worked independently, and if applicable, details of automation tools used in the process. | p.9 |
| Effect measures | 12 | Specify for each outcome the effect measure(s) (e.g. risk ratio, mean difference) used in the synthesis or presentation of results. | p.8-9 |
| Synthesis methods | 13a | Describe the processes used to decide which studies were eligible for each synthesis (e.g. tabulating the study intervention characteristics and comparing against the planned groups for each synthesis (item #5)). | p.8-9 |
|  | 13b | Describe any methods required to prepare the data for presentation or synthesis, such as handling of missing summary statistics, or data conversions. | p.8-9 |
|  | 13c | Describe any methods used to tabulate or visually display results of individual studies and syntheses. | p.8-9 |
|  | 13d | Describe any methods used to synthesize results and provide a rationale for the choice(s). If meta-analysis was performed, describe the model(s), method(s) to identify the presence and extent of statistical heterogeneity, and software package(s) used. | p.8-9 |
|  | 13e | Describe any methods used to explore possible causes of heterogeneity among study results (e.g. subgroup analysis, meta-regression). | p.9 |
|  | 13f | Describe any sensitivity analyses conducted to assess robustness of the synthesized results. | N/A |
| Reporting bias assessment | 14 | Describe any methods used to assess risk of bias due to missing results in a synthesis (arising from reporting biases). | p.9 |
| Certainty assessment | 15 | Describe any methods used to assess certainty (or confidence) in the body of evidence for an outcome. | N/A |
| **RESULTS** | | |  |
| Study selection | 16a | Describe the results of the search and selection process, from the number of records identified in the search to the number of studies included in the review, ideally using a flow diagram. | p. 10 + Figure 1 |
|  | 16b | Cite studies that might appear to meet the inclusion criteria, but which were excluded, and explain why they were excluded. | Figure 1 |
| Study characteristics | 17 | Cite each included study and present its characteristics. | Table 1 |
| Risk of bias in studies | 18 | Present assessments of risk of bias for each included study. | Table 3 and 4 |
| Results of individual studies | 19 | For all outcomes, present, for each study: (a) summary statistics for each group (where appropriate) and (b) an effect estimate and its precision (e.g. confidence/credible interval), ideally using structured tables or plots. | Table 2 + Figure 2 |
| Results of syntheses | 20a | For each synthesis, briefly summarise the characteristics and risk of bias among contributing studies. | p.12-15 |
|  | 20b | Present results of all statistical syntheses conducted. If meta-analysis was done, present for each the summary estimate and its precision (e.g. confidence/credible interval) and measures of statistical heterogeneity. If comparing groups, describe the direction of the effect. | p.12-15 |
|  | 20c | Present results of all investigations of possible causes of heterogeneity among study results. | p.15 |
|  | 20d | Present results of all sensitivity analyses conducted to assess the robustness of the synthesized results. | N/A |
| Reporting biases | 21 | Present assessments of risk of bias due to missing results (arising from reporting biases) for each synthesis assessed. | Appendix 6 |
| Certainty of evidence | 22 | Present assessments of certainty (or confidence) in the body of evidence for each outcome assessed. | N/A |
| **DISCUSSION** | | |  |
| Discussion | 23a | Provide a general interpretation of the results in the context of other evidence. | p.19-25 |
|  | 23b | Discuss any limitations of the evidence included in the review. | p.25-27 |
|  | 23c | Discuss any limitations of the review processes used. | p.25-27 |
|  | 23d | Discuss implications of the results for practice, policy, and future research. | p.24-27 |
| **OTHER INFORMATION** | | |  |
| Registration and protocol | 24a | Provide registration information for the review, including register name and registration number, or state that the review was not registered. | p.6 |
|  | 24b | Indicate where the review protocol can be accessed, or state that a protocol was not prepared. | p.6 |
|  | 24c | Describe and explain any amendments to information provided at registration or in the protocol. | N/A |
| Support | 25 | Describe sources of financial or non-financial support for the review, and the role of the funders or sponsors in the review. | p.29 |
| Competing interests | 26 | Declare any competing interests of review authors. | p.29 |
| Availability of data, code and other materials | 27 | Report which of the following are publicly available and where they can be found: template data collection forms; data extracted from included studies; data used for all analyses; analytic code; any other materials used in the review. | p.29 |

*From:*  Page MJ, McKenzie JE, Bossuyt PM, Boutron I, Hoffmann TC, Mulrow CD, et al. The PRISMA 2020 statement: an updated guideline for reporting systematic reviews. BMJ 2021;372:n71. doi: 10.1136/bmj.n71

**Appendix 2**

**Table S2** Search strategy of the literature

| **MEDLINE**  Date of search: 17.12.2022   \| - S41 \| S9 AND S40 \| \| --- \| --- \| \| - S40 \| S26 OR S39 \| \| - S39 \| S27 OR S28 OR S29 OR S30 OR S31 OR S32 OR S33 OR S34 OR S35 OR S36 OR S37 OR S38 \| \| - S38 \| TX Retrospective \| \| - S37 \| MH Cross-sectional studies \| \| - S36 \| TX Cross sectional \| \| - S35 \| TX Longitudinal \| \| - S34 \| TX (observational N1 (study or studies)) \| \| - S33 \| TX (Follow up N1 (study or studies)) \| \| - S32 \| TX Cohort analy* \| \| - S31 \| TX (cohort N2 (study or studies)) \| \| - S30 \| TX Case control \| \| - S29 \| MH cohort studies \| \| - S28 \| MH Case-Control Studies \| \| - S27 \| MH Epidemiologic studies \| \| - S26 \| S21 NOT S25 \| \| - S25 \| S22 OR S23 OR S24 \| \| - S24 \| MH historical article \| \| - S23 \| MH letter \| \| - S22 \| TX case report \| \| - S21 \| S13 OR S20 \| \| - S20 \| S14 OR S15 OR S16 OR S17 OR S18 OR S19 \| \| - S19 \| TX (allocated N2 random*) \| \| - S18 \| TX randomly allocated \| \| - S17 \| TX placebo* \| \| - S16 \| MH PLACEBOS \| \| - S15 \| TX ((singl? or doubl? or treb* or tripl*) N1 (blind* or mask*)) \| \| - S14 \| TX clinical N3 trial? \| \| - S13 \| S10 OR S11 OR S12 \| \| - S12 \| MH Clinical Trials as topic \| \| - S11 \| PT clinical trial, phase i OR clinical trial, phase ii OR clinical trial, phase iii OR clinical trial, phase iv OR controlled clinical trial OR randomized controlled trial OR multicenter study OR clinical trial \| \| - S10 \| MH Randomized Controlled Trials as Topic OR MH randomized controlled trial OR MH Random Allocation OR MH Double Blind Method OR MH Single Blind Method OR MH clinical trial \| \| - S9 \| S4 AND S8 \| \| - S8 \| S5 OR S6 OR S7 \| \| - S7 \| TI ability to work OR work abilit* OR capacity to work OR work* capacity OR work outcome* OR work participation OR labo?r participation OR employabil* OR productivity OR workplace OR work place* OR return to work OR return-to-work OR back to work OR absenteeism OR sickness absence OR sick leave OR disability leave OR occupational health OR reintegration OR re-integration \| \| - S6 \| AB ability to work OR work abilit* OR capacity to work OR work* capacity OR work outcome* OR work participation OR labo?r participation OR employabil* OR productivity OR workplace OR work place* OR return to work OR return-to-work OR back to work OR absenteeism OR sickness absence OR sick leave OR disability leave OR occupational health OR reintegration OR re-integration \| \| - S5 \| MH workplace OR MH return to work OR MH absenteeism OR MH occupational health OR MH work performance OR MH work capacity evaluation OR sick leave \| \| - S4 \| S1 OR S2 OR S3 \| \| - S3 \| TI covid OR covid-19 OR coronavirus* OR corona virus* OR 2019-ncov OR sars-cov-2 OR cov-19 OR covid 19 OR covid19 OR severe acute respiratory syndrome coronavirus 2 OR coronavirus infection* Or covid infection* OR post COVID-19 OR post-acute COVID-19 OR post-acute COVID-19 syndrome \| \| - S2 \| AB covid OR covid-19 OR coronavirus* OR corona virus* OR 2019-ncov OR sars-cov-2 OR cov-19 OR covid 19 OR covid19 OR severe acute respiratory syndrome coronavirus 2 OR coronavirus infection* OR covid infection* OR post COVID-19 OR post-acute COVID-19 OR post-acute COVID-19 syndrome \| \| - S1 \| MH coronavirus OR MH covid-19 OR MH sars-cov-2 OR MH coronavirus infections OR MH betacoronavirus \|   Results: **1.507**  **EMBASE**  Date of search: 17.12.2022   1. exp Coronavirinae/ or exp coronavirus disease 2019/ or exp Coronavirus infection/ or exp Severe acute respiratory syndrome coronavirus 2/ or exp Betacoronavirus/ 2. (covid or covid-19 or coronavirus* or corona virus* or 2019-ncov or sars-cov-2 or cov-19 or covid 19 or covid19 or severe acute respiratory syndrome coronavirus 2 or post COVID-19 or post-acute COVID-19 or post-acute COVID-19 syndrome or coronavirus infection*).ab,ti. 3. 1 or 2 4. work capacity/ or workplace/ or return to work/ or employment/ or absenteeism/ or occupational health/ or job performance/ or medical leave/ 5. (ability to work or work abilit* or capacity to work or work* capacity or work outcome* or work participation or labo?r participation or employabil* or productivity or workplace or work place* or return to work or return-to-work or back to work or absenteeism or sickness absence or sick leave or disability leave or occupational health or reintegration or re-integration).ab,ti. 6. 4 or 5 7. 3 and 6 8. Clinical Trial/ 9. Randomized Controlled Trial/ 10. controlled clinical trial/ 11. multicenter study/ 12. Phase 3 clinical trial/ 13. Phase 4 clinical trial/ 14. exp RANDOMIZATION/ 15. Single Blind Procedure/ 16. Double Blind Procedure/ 17. Crossover Procedure/ 18. PLACEBO/ 19. randomi?ed controlled trial$.tw. 20. rct.tw. 21. (random$ adj2 allocat$).tw. 22. single blind$.tw. 23. double blind$.tw. 24. ((treble or triple) adj blind$).tw. 25. placebo$.tw. 26. Prospective Study/ 27. 8 or 9 or 10 or 11 or 12 or 13 or 14 or 15 or 16 or 17 or 18 or 19 or 20 or 21 or 22 or 23 or 24 or 25 or 26 28. Case Study/ 29. case report.tw. 30. abstract report/ or letter/ 31. Conference proceeding.pt. 32. Conference abstract.pt. 33. Editorial.pt. 34. Letter.pt. 35. Note.pt. 36. 28 or 29 or 30 or 31 or 32 or 33 or 34 or 35 37. 27 not 36 38. Clinical study/ 39. Case control study/ 40. Family study/ 41. Longitudinal study/ 42. Retrospective study/ 43. Prospective study/ 44. Randomized controlled trials/ 45. 43 not 44 46. Cohort analysis/ 47. (Cohort adj (study or studies)).mp. 48. (Case control adj (study or studies)).tw. 49. (follow up adj (study or studies)).tw. 50. (observational adj (study or studies)).tw. 51. (epidemiologic$ adj (study or studies)).tw. 52. (cross sectional adj (study or studies)).tw. 53. 38 or 39 or 40 or 41 or 42 or 45 or 46 or 47 or 48 or 49 or 50 or 51 or 52 54. 37 or 53 55. 7 and 54   Results: **1.769**  **CINAHL**  Date of search: 17.12.2022   \| - S32 \| S9 AND S30 \| \| --- \| --- \| \| - S31 \| S9 AND S30 \| \| - S30 \| S21 OR S29 \| \| - S29 \| S22 OR S23 OR S24 OR S25 OR S26 OR S27 OR S28 \| \| - S28 \| TX observational N2 (study or studies) \| \| - S27 \| TX cohort N2 (study or studies) \| \| - S26 \| MH cross sectional stud* \| \| - S25 \| MH Nonconcurrent prospective stud* \| \| - S24 \| MH correlational stud* \| \| - S23 \| MH case control stud* \| \| - S22 \| MH prospective stud* \| \| - S21 \| S10 OR S11 OR S12 OR S13 OR S14 OR S15 OR S16 OR S17 OR S18 OR S19 OR S20 \| \| - S20 \| TX allocat* random* \| \| - S19 \| (MH "Quantitative Studies") \| \| - S18 \| (MH "Placebos") \| \| - S17 \| TX placebo* \| \| - S16 \| TX random* allocat* \| \| - S15 \| (MH "Random Assignment") \| \| - S14 \| TX randomi* control* trial* \| \| - S13 \| ( TX ( (singl* n1 blind*) or (singl* n1 mask*) ) ) OR ( TX ( (doubl* n1 blind*) or (doubl* n1 mask*) ) ) OR ( TX ( (tripl* n1 blind*) or (tripl* n1 mask*) ) ) OR ( TX ( (trebl* n1 blind*) or (trebl* n1 mask*) ) ) \| \| - S12 \| TX clinic* n1 trial* \| \| - S11 \| PT Clinical trial \| \| - S10 \| (MH "Clinical Trials+") \| \| - S9 \| S4 AND S8 \| \| - S8 \| S5 OR S6 OR S7 \| \| - S7 \| TI ability to work OR work abilit* OR capacity to work OR work* capacity OR work outcome* OR work participation OR labo?r participation OR employabil* OR productivity OR workplace OR work place* OR return to work OR return-to-work OR back to work OR absenteeism OR sickness absence OR sick leave OR disability leave OR occupational health OR reintegration OR re-integration \| \| - S6 \| AB ability to work OR work abilit* OR capacity to work OR work* capacity OR work outcome* OR work participation OR labo?r participation OR employabil* OR productivity OR workplace OR work place* OR return to work OR return-to-work OR back to work OR absenteeism OR sickness absence OR sick leave OR disability leave OR occupational health OR reintegration OR re-integration \| \| - S5 \| MH work environment OR MH job re-entry OR MH absenteeism OR MH sick leave OR MH occupational health \| \| - S4 \| S1 OR S2 OR S3 \| \| - S3 \| TI covid OR covid-19 OR coronavirus* OR corona virus* OR 2019-ncov OR sars-cov-2 OR cov-19 OR covid 19 OR covid19 OR severe acute respiratory syndrome coronavirus 2 OR coronavirus infection* OR covid infection* OR post COVID-19 OR post-acute COVID-19 OR post-acute COVID-19 syndrome \| \| - S2 \| AB covid OR covid-19 OR coronavirus* OR corona virus* OR 2019-ncov OR sars-cov-2 OR cov-19 OR covid 19 OR covid19 OR severe acute respiratory syndrome coronavirus 2 OR coronavirus infection* OR covid infection* OR post COVID-19 OR post-acute COVID-19 OR post-acute COVID-19 syndrome \| \| - S1 \| MH coronavirus OR MH covid-19 OR MH sars-cov-2 OR MH coronavirus infections OR MH Post-Acute COVID-19 Syndrome \|   Results: **1.058**  **CENTRAL**  Date of search: 17.12.2022  #1 MeSH descriptor: [Coronavirus] explode all trees  #2 MeSH descriptor: [COVID-19] explode all trees  #3 MeSH descriptor: [SARS-CoV-2] explode all trees  #4 MeSH descriptor: [Coronavirus Infections] explode all trees  #5 MeSH descriptor: [Betacoronavirus] explode all trees  #6 (covid OR covid-19 OR coronavirus* OR corona virus* OR sars-cov-2 OR cov-19 OR covid 19 OR covid19 OR severe acute respiratory syndrome coronavirus 2 OR coronavirus infection* OR covid infection* OR post COVID-19 OR post-acute COVID-19 OR post-acute COVID-19 syndrome):ti,ab,kw  #7 #1 OR #2 OR #3 OR #4 OR #5 OR #6  #8 MeSH descriptor: [Workplace] explode all trees  #9 MeSH descriptor: [Return to Work] explode all trees  #10 MeSH descriptor: [Absenteeism] explode all trees  #11 MeSH descriptor: [Sick Leave] explode all trees  #12 MeSH descriptor: [Work Capacity Evaluation] explode all trees  #13 MeSH descriptor: [Occupational Health] explode all trees  #14 MeSH descriptor: [Work Performance] explode all trees  #15 (ability to work OR work abilit* OR capacity to work OR work* capacity OR work outcome* OR work participation OR labo?r participation OR employabil* OR productivity OR workplace OR work place* OR return to work OR return-to-work OR back to work OR absenteeism OR sickness absence OR sick leave OR disability leave OR occupational health OR reintegration OR re-integration):ti,ab,kw  #16 #8 OR #9 OR #10 OR #11 OR #12 OR #13 OR #14 OR #15  #17 #7 AND #16 with Publication Year from 2020 to 2022, in Trials  Results: **729**  **WHO COVID-19**  Date of search: 17.12.2022   - (ti:(((ability TO work) OR (work ability) OR (capacity TO work) OR (work participation) OR (work performance) OR (employability) OR (return TO work) OR (return-to-work) OR (back TO work) OR (occupational health) OR (sick leave) OR (reintegration)) )) OR - (ab:(((ability TO work) OR (work ability) OR (capacity TO work) OR (work participation) OR (work performance) OR (employability) OR (return TO work) OR (return-to-work) OR (back TO work) OR (occupational health) OR (sick leave) OR (reintegration)) )) AND - type_of_study:("prognostic_studies" OR "observational_studies" OR "experimental_studies" OR "rct" OR "cohort_studies" OR "diagnostic_studies") AND - covidwho_topics:("long_covid") AND - la:("en" OR "de")   Results: **904** |
| --- | --- | --- | --- | --- | --- | --- | --- | --- | --- | --- | --- | --- | --- | --- | --- | --- | --- | --- | --- | --- | --- | --- | --- | --- | --- | --- | --- | --- | --- | --- | --- | --- | --- | --- | --- | --- | --- | --- | --- | --- | --- | --- | --- | --- | --- | --- | --- | --- | --- | --- | --- | --- | --- | --- | --- | --- | --- | --- | --- | --- | --- | --- | --- | --- | --- | --- | --- | --- | --- | --- | --- | --- | --- | --- | --- | --- | --- | --- | --- | --- | --- | --- | --- | --- | --- | --- | --- | --- | --- | --- | --- | --- | --- | --- | --- | --- | --- | --- | --- | --- | --- | --- | --- | --- | --- | --- | --- | --- | --- | --- | --- | --- | --- | --- | --- | --- | --- | --- | --- | --- | --- | --- | --- | --- | --- | --- | --- | --- | --- | --- | --- | --- | --- | --- | --- | --- | --- | --- | --- | --- | --- | --- | --- | --- | --- | --- |

**Appendix 3**

**Table S3** Post-COVID-symptoms

| Authors | post-COVID-symptoms |
| --- | --- |
| Amorim et al. (2022) | body aches (44%), weakness (41%), headache (38%), shortness of breath (26%), dizziness (25%) |
| Davis et al. (2021) | fatigue (85%), post-exertional malaise (76.4%), brain fog (66.7%), shortness of breath (56.4%), memory issues (54.3%) |
| Delgado-Alonso et al. (2022) | cognitive issues (92.2%), fatigue (84.4%), headache (76.6%), sleep disorders (70.1%), weakness (66.2%) |
| Diem et al. (2022) | fatigue (86%), pain including headache (66.1%), sleep disturbances (44.9%), dizziness (41.8%), dyspnea (38.7%) |
| Hodgson et al. (2021) | shortness of breath (34.8%), loss of strength (21.7%), fatigue (19.1%), persistent cough (13.9%), loss of taste (12.2%) |
| Kedor et al. (2022) | fatigue (100%), post-exertional malaise (100%), impaired performance (96%), stress intolerance (96%), Concentration impairment (91%) |
| Kisiel et al. (2022) | fatigue (34%), impaired taste/smell (28%), dyspnea (21%), memory/concentration problems (14%), worsening physical/mental activity (13%) |
| Müller et al. (2023) | exercise intolerance (100%), neurological ailments (98%), fatigue (91%), chest pain (90%), sleep disturbance (83%) |
| Peters et al. (2022) | fatigue/exhaustion (82.9%), concentration/memory problems (70.7%), shortness of breath (56.5%), headache (41.3%), loss of taste/smell (38.1%) |
| Rutsch et al. (2022) | shortness of breath during exertion (71%), tiredness (68%), lack of strength (63%), exhaustion (63%), problems with concentration (57%) |
| Sansone et al. (2022) | fatigue (88.5%), shortness of breath (75.4%), myalgia (55.7%), arthralgia (54.9%), headache (47.5%) |
| van Wambeke et al. (2023) | fatigue (>90%), neurocognitive disorders (>70%), muscle and joint pain (>60%), dyspnea (>30%), taste and smell (>20%) |
| Wahlgren et al. (2023) | difficulty being active (45%), fatigue (41%), weakness (41%), remembering (40%), stress (39%) |
| Note: The prevalence rates provided in the table are based on the respective studies and may not be directly comparable due to differences in sample sizes, study populations, and methodologies. | |

**Appendix 4**

**Table S4.1** Modified Newcastle-Ottawa Quality Assessment Scale criteria for cohort studies

| **Cohort Studies**  *Note*: A study can be awarded a maximum of one star (★) for each numbered item within the Selection and Outcome categories. A maximum of two stars (★★) can be given for Comparability.  **Maximum**: 9 stars  **Methodological Quality Rank**: high=9-7 ★, moderate=6-5 ★, low=4 or fewer ★   \| **Selection** \| \| \| --- \| --- \| \| *1. Representativeness of the exposed cohort [tested/clinically diagnosed COVID-19 positive]* \| - 1. truly representative of target population (e.g., nation-wide database) ★   2. somewhat representative of target population (e.g., city, hospital/hospital system, social media survey) ★   3. selected groups of participants (i.e. subgroups: sex, occupation, disease severity, pre-existing condition). Restricting inclusion criteria to adults does not count as a subgroup.   4. no description of the derivation of the cohort \| \| *2. Selection of the non-exposed cohort [tested COVID-19 negative/never tested COVID-19 positive or suspected to have had COVID-19]* \| - 1. drawn from the same community/database/hospital as the exposed cohort ★   2. drawn from a different source   3. no description of the derivation of the non-exposed cohort   4. no non-exposed cohort included \| \| *3. Ascertainment of exposure [COVID-19]* \| 1. secure medical/hospital records or validated measurement tool (laboratory testing) ★ 2. diagnosis based upon clinical judgment ★ 3. self-report of test positivity 4. no description \| \| *4. Demonstration that outcome of interest was not present at start of study* \| 1. yes ★ 2. no \|  \| **Comparability** \| \| \| --- \| --- \| \| *1. Comparability of cohorts on the basis of the design or analysis* \| 1. study controls and/or regression analysis for age or sex ★ 2. study controls and/or regression analysis for any additional factor (e.g., co-morbidities, occupation, COVID-19 severity) ★ \|  \| **Outcome** \| \| \| --- \| --- \| \| *1. Assessment of outcome [work ability, return-to-work]* \| 1. validated objective assessment tool (e.g., work ability index (WAI)) for at least 1 outcome of interest ★ 2. structured/systematic interview or questionnaire conducted by trained healthcare or research professional ★ 3. unstructured self-report (i.e., open question regarding work ability, return-to-work) and/or not conducted by trained healthcare or research professional (i.e., self-administered) or not stated 4. no description \| \| *2. Was follow-up long enough for outcomes to occur?* \| - 1. yes (≥12 weeks from diagnosis of COVID-19) ★   2. no (≤12 weeks) \| \| *3. Adequacy of follow up of cohorts* \| - 1. complete follow up; all subjects accounted for ★   2. subjects lost to follow up unlikely to introduce bias: ≤20% of initial sample size lost, or description provided of those lost ★   3. lost **>**20% of initial sample size during follow up, and no description of those lost   4. no statement \| |
| --- | --- | --- | --- | --- | --- | --- | --- | --- | --- | --- | --- | --- | --- | --- | --- | --- | --- | --- | --- | --- | --- | --- |

**Table S4.2** Modified Newcastle-Ottawa Quality Assessment Scale criteria for cross-sectional studies

| **Cross-sectional Studies**  *Note*: A study can be awarded a maximum of one star (*) for items 1-3 for Selection and the Outcome categories.  A maximum of two stars (**) can be given for item 4 in Selection, and Comparability.  **Maximum**: 9 stars  **Methodological Quality Rank**: high=9-7 ★, moderate=6-5 ★, low=4 or fewer ★   \| **Selection** \| \| \| --- \| --- \| \| *1. Representativeness of the sample* \| - 1. truly representative of the average in the target population (all subjects or random sampling) ★   2. somewhat representative of the average in the target population (non-random sampling) ★   3. selected group of users   4. no description of the sampling strategy \| \| *2. Sample size* \| - 1. justified and satisfactory (including pre-determined sample size calculation) ★   2. not justified (not pre-determined through calculation)   3. no information provided \| \| *3. Non-respondents* \| 1. comparability between respondents and non-respondents characteristics is established, and the response rate is satisfactory (>60%) or proportion of target sample recruited attains pre-specified target ★ 2. unsatisfactory recruitment rate, no summary data on non-respondents, or the comparability between respondents and non-respondents is unsatisfactory 3. no description of the response rate or the characteristics of the responders and non-responders \| \| *4. Ascertainment of the exposure [COVID-19]* \| 1. secure medical/hospital records indicative of test positivity or validated measurement tool (laboratory testing) ★★ 2. diagnosis based upon clinical judgment ★ 3. self-report of test positivity 4. no description \|  \| **Comparability** \| \| \| --- \| --- \| \| *1. Comparability of subjects in different outcome groups on the basis of design or analysis* \| 1. data/ results controlled for age or sex, or separate analyses reported for each age group or sex ★ 2. data/results controlled for any additional factor (e.g., comorbidities), or separate analyses reported for any additional factor ★ \|  \| **Outcome** \| \| \| --- \| --- \| \| *1. Assessment of the outcome [work ability, return-to-work]* \| 1. validated objective assessment tool (e.g., work ability index (WAI)) for at least 1 outcome of interest ★ 2. structured/systematic interview or questionnaire conducted by trained healthcare or research professional ★ 3. unstructured self-report (i.e., open question regarding work ability, return-to-work) and/or not conducted by trained healthcare or research professional (i.e., self-administered) or not stated 4. no description \| \| *2. Statistical methodology* \| 1. statistical test used to analyse the data clearly described and appropriate ★ 2. statistical test not appropriate, not described or incomplete \| |
| --- | --- | --- | --- | --- | --- | --- | --- | --- | --- | --- | --- | --- | --- | --- | --- | --- | --- | --- | --- | --- |

**Appendix 5**


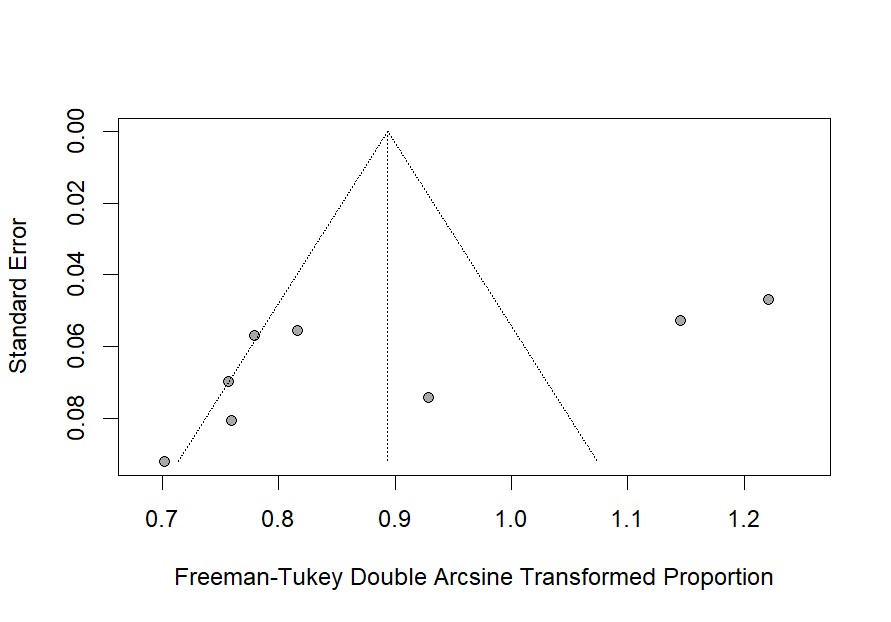


**Figure S1** Funnel plot
